# Exploring the DNA_2_-PNA heterotriplex formation in targeting the Bcl-2 gene promoter: A structural insight by physico-chemical and microsecond-scale MD investigation

**DOI:** 10.1016/j.heliyon.2024.e24599

**Published:** 2024-01-22

**Authors:** Andrea P. Falanga, Antonio Lupia, Lorella Tripodi, Carmine M. Morgillo, Federica Moraca, Giovanni N. Roviello, Bruno Catalanotti, Jussara Amato, Lucio Pastore, Vincenzo Cerullo, Stefano D'Errico, Gennaro Piccialli, Giorgia Oliviero, Nicola Borbone

**Affiliations:** a Dipartimento di Farmacia, Università Degli Studi di Napoli Federico II, Naples, 80131, Italy; b Dipartimento di Medicina Molecolare e Biotecnologie Mediche, Università Degli Studi di Napoli Federico II, Naples, 80131, Italy; c CEINGE-Biotecnologie Avanzate Franco Salvatore S.c.a.r.l., Naples, 80145, Italy; d Istituto di Biostrutture e Bioimmagini, Consiglio Nazionale Delle Ricerche, Naples, 80131, Italy; e ImmunoViroTherapy Lab (IVT), Drug Research Program (DRP), Faculty of Pharmacy, University of Helsinki, 00100, Helsinki, Finland

**Keywords:** PNA anti-Bcl-2, PNA gene expression modifiers, PNA-adenoviral vector, Bcl-2 gene modulation, Drug delivery, Ad5Delta24 (OAd)

## Abstract

Peptide Nucleic Acids (PNAs) represent a promising tool for gene modulation in anticancer treatment. The uncharged peptidyl backbone and the resistance to chemical and enzymatic degradation make PNAs highly advantageous to form stable hybrid complexes with complementary DNA and RNA strands, providing higher stability than the corresponding natural analogues. Our and other groups’ research has successfully shown that tailored PNA sequences can effectively downregulate the expression of human oncogenes using antigene, antisense, or anti-miRNA approaches. Specifically, we identified a seven bases-long PNA sequence, complementary to the longer loop of the main G-quadruplex structure formed by the bcl2midG4 promoter sequence, capable of downregulating the expression of the antiapoptotic Bcl-2 protein and enhancing the anticancer activity of an oncolytic adenovirus. Here, we extended the length of the PNA probe with the aim of including the double-stranded Bcl-2 promoter among the targets of the PNA probe. Our investigation primarily focused on the structural aspects of the resulting DNA_2_-PNA heterotriplex that were determined by employing conventional and accelerated microsecond-scale molecular dynamics simulations and chemical-physical analysis. Additionally, we conducted preliminary biological experiments using cytotoxicity assays on human A549 and MDA-MB-436 adenocarcinoma cell lines, employing the oncolytic adenovirus delivery strategy.

## 1 Introduction

Peptide Nucleic Acids (PNAs) represent one of the most promising candidates among the reported antigene tools to modulate gene activity. PNAs are DNA analogues in which N-(2-aminoethyl)glycine units replace the natural sugar-phosphate backbone. Usually, PNAs interact with single-stranded complementary RNA and DNA oligonucleotides to form Watson-Crick hydrogen-paired DNA-PNA heteroduplexes that are more stable than the corresponding RNA-DNA or DNA-DNA counterparts [1]. PNAs are also capable of behaving as G-quadruplex binders [2] or triplex-forming (TF) agents [3,4] depending on their length, sequence, and the structural features of the DNA or RNA target. These remarkable properties, together with the higher chemical and enzymatic stability and lower toxicity [5], render PNAs suitable molecular tools in biosensing [6,7] and biomedical applications using the antigene [2,8], antisense [9,10], antimiRNA [[11], [12], [13], [14], [15]], and gene editing [4,16] strategies. Several anticancer approaches based on PNA technology have been reported [9,10,17]. In this context, we studied the ability of PNA oligomers to target the loops of DNA G-quadruplexes (G4s) formed in the promoters of human oncogenes [2,18] aiming at downregulating their expression. In particular, we demonstrated that the 7-mer PNA having sequence ccttcct (N-to-C) complementary to the longest loop of the G-quadruplex structure formed by the 23-mer G-rich DNA sequence GGGCGCGGGAGGAAGGGGGCGGG (bcl2midG4_G, Table 1) located in the P1 promoter of the B-cell lymphoma 2 oncogene (Bcl-2) selectively binds its G4 target over the corresponding duplex tract (bcl2midG4-ds, Table 1) and downregulates the expression of the Bcl-2 gene in human A549 and MDA-MB-436 cancer cell lines [18]. The product of the Bcl-2 gene is a mitochondrial protein responsible for regulating programmed cell death. Because of its role as an inhibitor of apoptosis, elevated levels of Bcl-2 expression have been detected in various human cancers such as breast, cervical, non-small cell lung, and prostate [[19], [20], [21]]. Furthermore, the oncogenic capabilities of the Bcl-2 gene can be attributed in part to its role in promoting resistance to chemotherapy-induced apoptosis [22]. The development of PNA-based drugs has been so far limited by the poor cell permeability of PNAs. In our previous study, we used the oncolytic adenovirus Ad5Delta24 (OAd) as the PNA drug delivery system considering its ability to selectively infect and kill cancer cells over non-proliferating cells, because of the 24bp deletion in its E1A gene that strongly reduces the virus replication in non-tumoral cells [23,24], as well as to specifically deliver in the latter any molecule adsorbed on the OAd capsid thus exerting a synergistic anticancer outcome [25]. On this ground, aiming at increasing the target specificity of the PNA probe towards the Bcl-2 P1 promoter and at overcoming the unfavourable ratio between the G4-folded DNA target and the corresponding duplex tract, we decided to extend the PNA probe length from seven to ten bases, thus obtaining the 10-mer triplex-forming PNA, TF-PNA1K (Table 1), whose interaction with the complementary bcl2midG4_G target, either structured in its duplex or G-quadruplex form, was studied by circular dichroism (CD) and gel electrophoresis (PAGE). To confer the water solubility required for this study, one lysine residue (K) was appended at the C-end of TF-PNA1K. Given the homopyrimidine sequence of TF-PNA1K, the new PNA was designed to form a stable DNA_2_-PNA heterotriplex (referred to as “triplex” in Table 1) with the target bcl2midG4-ds [26,27], though preserving the ability to bind the G4 structure formed by the bcl2midG4_G sequence. In addition, a computational study was performed on the DNA_2_-PNA complex to get insight into the structural details of this unusual triplex. As reported, the formation of stable DNA-PNA heterotriplexes in gene promoters downregulates genes expression by hindering the DNA unwinding, the binding of transcription factors, and the elongation of mRNAs by RNA polymerase [28]. Although the mechanistic aspects of DNA-PNA triplexes formation are quite well known [29], more detailed structural information on the DNA-PNA heterotriplexes is still needed. Here, we discuss the physico-chemical characterization of the complexes formed when TF-PNA1K, complementary to the N_10–19_ tract of bcl2midG4_G sequence, is incubated with the target DNA sequences and report the structural details of the resulting DNA_2_-PNA heterotriplex as assessed by unbiased molecular dynamics calculations. The effect on cell viability of human A549 and MDA-MB-436 adenocarcinoma cell lines incubated with bare and TF-PNA6K-coated Ad5Delta24 OAd is also reported.

**Table 1:** Sequences of DNA and PNA molecules used in this study. Upper case letters indicate DNA bases, lower case letters indicate PNA bases, plain bonds indicate Watson-Crick pairing, and dashed bonds indicate reverse Hoogsteen pairing.

Sample	Sequence
bcl2midG4_G d (5ʹ→3ʹ)	GGGCGCGGGAGGAAGGGGGCGGG
bcl2midG4_C d (5ʹ→3ʹ)	CCCGCCCCCTTCCTCCCGCGCCC
TF-PNA1K (N→C)	cccccttcct-K
TF-PNA6K (N→C)	cccccttcct-KKKKKK
bcl2midG4-ds/TF-PNA1K (triplex)	

## 2 Materials and methods

### 2.1 Synthesis and analysis of DNA samples

The bcl2midG4_G oligonucleotide (ON) and its complementary C-rich strand (bcl2midG4_C, see Table 1) were obtained through solid-phase β-cyanoethyl phosphoramidite chemistry employing an Expedite 8909 automated DNA synthesizer (Perseptive biosystem, Framingham, MA, USA). The syntheses occurred on a CPG 10.13039/100016549Universal Support (35 mg, 1.4 μmol) using a standard 1 μmol scale protocol with the DMT-OFF option. Post-synthesis, the oligomers were released from the support and deprotected by treating them with concentrated aqueous ammonia at 55 °C for 12 h. The combined filtrates and washings were concentrated under reduced pressure, re-dissolved in H_2_O, and purified by HPLC using a Jasco system (PU2089 Plus quaternary pump equipped with the Jasco 2075 Plus UV detector; Jasco Europe S.r.l., Cremella, Italy) with a Nucleogel 1000–8/46 SAX column (Macherey-Nagel, Düren, Germany) which was eluted with a linear gradient of buffer B (1.0 mol/L NaCl and 20 mmol/L NaH_2_PO_4_ aqueous solution containing 20 % acetonitrile (v/v), pH 7.0) in buffer A (20 mmol/L NaH_2_PO_4_ aqueous solution containing 20 % acetonitrile (v/v), pH 7.0), from 0 % to 100 %, in 30 min at a flow rate of 1.2 mL/min. The ONs were collected and subsequently desalted using C18 Sep-Pak cartridges (Waters Italia, Sesto San Giovanni, Italy) eluted with a stepped gradient of acetonitrile in H_2_O (30, 50, and 100 %). The isolated ONs exhibited >99 % purity (NMR, data not shown). The ON concentration was determined spectrophotometrically at λ = 260 nm and 90 °C, using the molar extinction coefficient *ε* = 231 300 for bcl2midG4_G and *ε* = 175 400 L/(cm mol) for bcl2midG4_C, as determined via the Sigma-Aldrich OligoEvaluatorTM web tool (www.oligoevaluator.com).

### 2.2 Synthesis and analysis of PNA samples

TF-PNA1K and TF-PNA6K (Table 1) were synthesized employing the 9-fluorenylmethoxycarbonyl (Fmoc) solid-phase strategy. Initially, 50 mg of 4-methylbenzhydrylamine (MBHA) resin (0.5 mmol/g) underwent swelling in dimethylformamide (DMF) overnight. Subsequently, the resin was treated with a solution of 20 % piperidine in DMF for 10 min. After DMF washes (repeated five times), either one or six lysine couplings were carried out using Fmoc-L-Lys (MMt)-OH (MMT = monomethoxytrityl) under the following conditions: 2.5 equivalents of a 0.2 mol/L solution of Fmoc-L-Lys monomer in N-methyl-2-pyrrolidone (NMP), 2.5 equivalents of a 0.2 mol/L solution of hexafluorophosphate azabenzotriazole tetramethyl uronium (HATU) in DMF, 2.5 equivalents of N,N-diisopropylethylamine (DIPEA), and 3.75 equivalents of lutidine for 40 min at room temperature (RT). PNA monomers were then introduced using the following conditions: 5 equivalents of a 0.2 mol/L solution of the monomer building block in NMP, 5 equivalents of a 0.2 mol/L solution of HATU in DMF, 5 equivalents of DIPEA, and 7.5 equivalents of lutidine for 40 min at RT. After each coupling step, a capping with Ac_2_O in the presence of DIPEA was performed for 10 min at RT, followed by removal of the Fmoc group through two treatments with a solution of 20 % piperidine in DMF for 10 min. At the conclusion of the synthetic cycle, the PNAs were detached from the solid support by treatment with trifluoroacetic acid (TFA)/anisole/ethanedithiol (9:1:1; v/v/v) for 4 h, and the products were precipitated with cold diethyl ether. The precipitates were recovered by centrifugation, washed twice with diethyl ether, dissolved in water, and finally lyophilized. The PNAs were obtained with a 48–50 % overall yield (95 % medium yield for each coupling estimated by Fmoc spectrophotometric measurements). The crude samples underwent purification through semipreparative HPLC analyses (Supplementary Fig. S1) on the aforementioned Jasco system, utilizing a Macherey-Nagel 10 × 250 mm C-18 reverse-phase column (particle size 5 μm). Elution was carried out with a linear gradient of acetonitrile containing 0.1 % (v/v) TFA in H_2_O containing 0.1 % (v/v) TFA (from 0 to 100 % in 45 min, flow 1.2 mL/min). Subsequently, the collected fractions were lyophilized, and the quantity of each PNA dissolved in pure water was estimated through quantitative UV with a Jasco V-530 spectrophotometer (Jasco Europe S.r.l., Cremella, Italy) (λ = 220–310 nm, 400 nm/min scanning speed, 2.0 nm bandwidth) using the molar extinction coefficient *ε* = 72 600 L/(cm mol) for both TF-PNA1K and TF-PNA6K. The final PNA products were characterized by ESI-MS on an ABSciex 4000 Q TRAP mass spectrometer (ThermoFisher Scientific, Waltham, MA, USA) operating in positive ion mode by preparing 20 μmol/L PNA solutions containing 0.1 % (v/v) formic acid in H_2_O (Supplementary Figs. S2 and S3).

### 2.3 Preparation of bcl2midG4-G4 and bcl2midG4-ds and their incubation with TF-PNA1K

All oligonucleotides were prepared at a concentration of 20 μmol/L in a 60 mmol/L potassium buffer, obtained by diluting a 1.0 mol/L buffered solution (comprising 100 mmol/L KH_2_PO_4_, 900 mmol/L KCl, with pH values of 7.0 or 5.0). For the preparation of bcl2midG4-G4, 10 nmol of bcl2midG4_G were lyophilized and reconstituted in 0.5 mL of 60 mmol/L potassium buffer to achieve the desired 20 μmol/L concentration. The solution underwent a thermodynamic annealing process, involving heating at 90 °C for 5 min followed by cooling to room temperature over a period of 12 h, to induce the G-quadruplex folding. The bcl2midG4-ds duplex was formed by dissolving 10 nmol of lyophilized bcl2midG4_G in 10 μL of a 1.0 mmol/L bcl2midG4_C stock solution. The resulting solution was then diluted to 0.5 mL with the 60 mmol/L potassium buffer at pH 7.0 or 5.0 and subjected to the same annealing procedure as described earlier. Both bcl2midG4-G4 and bcl2midG4-ds structures were equilibrated at 4 °C for a minimum of 4 h before the addition of TF-PNA1K at a 1:5 ratio of DNA secondary structure to PNA. The mixtures were then incubated at 4 °C for 24 h before data acquisition.

### 2.4 Circular dichroism (CD) studies

CD spectra were acquired using a Jasco 1500 spectropolarimeter coupled with a Jasco PTC-348-WI temperature controller (Jasco Europe S.r.l., Cremella, Italy) spanning the 200–320 nm range using 0.1-cm path length cuvettes. All samples were dissolved in the above-reported 60 mmol/L K^+^ buffers (pH values of 7.0 or 5.0) at the final concentration of 20 μmol/L. All CD spectra were recorded with a scan rate of 200 nm/min, a response time of 4 s, a bandwidth of 2 nm, and averaged over five scans. The buffer baseline was subtracted from each spectrum, and the spectra were normalized to exhibit zero ellipticity at 320 nm. Thermal denaturation experiments were conducted over the temperature range of 5–90 °C employing a heating rate of 0.5 °C/min and monitoring the following CD values: 263 nm for bcl2midG4-G4; 268 nm for the 1:5 bcl2midG4-G4/TF-PNA1K complex; 265 nm for the bcl2midG4-ds at pH 7.0 and 5.0; 268 nm and 276 nm for the bcl2midG4-ds/TF-PNA1K triplex at pH 7.0 and pH 5.0, respectively. CD spectra of all samples were acquired at 5, 50, and 90 °C during the heating and cooling runs.

### 2.5 Characterization by nondenaturing polyacrylamide gel electrophoresis (PAGE)

Nondenaturing polyacrylamide gel electrophoreses were performed using 18 % polyacrylamide gels which were run for 1 h at the constant voltage of 120 V, at 4 °C in 1 × Tris-Borate-EDTA (TBE) buffer supplemented with 30 mmol/L KCl (pH 7.0 or pH 5.0). All samples were loaded at a 20 μmol/L ON concentration. Before gel loading, 2 μL of each sample were added to 8 μL of loading buffer (glycerol/(1 × TBE + 30 mmol/L KCl) 1:9). The gels were stained with Sybr green (Sigma-Aldrich – Merck KGaA, Darmstadt, Germany) and visualized using a Gel Doc XR imaging system (Biorad, Milano, Italy).

### 2.6 Computational methods

#### 2.6.1 System preparation

The bcl2midG4 double helix (bcl2midG4-ds) was built as A-DNA and B-DNA using the 3D Dart web server [30]. Subsequently, these systems were submitted to 1 μs of molecular dynamics (MD) simulations. The representative structure of the main cluster of bcl2midG4-ds MD simulations was selected as the initial duplex structure for constructing the triplex system. The 3D structure of TF-PNA1K strand was extracted from a 13 bases long PNA reported in one of our previous works [13]. The strand was trimmed to include only the 10 central bases and mutated using the X3DNA mutate_bases tool [31] to match the correct sequence. Hence, the simulated bcl2midG4-ds and the PNA were aligned with the lowest energy NMR structure of the 7-mer DNA triplex (PDB ID: 149D) [32]. Finally, the PNA strand in Hoogsteen was manually adjusted to have a reasonable orientation of the complementary hydrogen bonds, and a lysine residue was added at the C-terminus of the PNA to complete the sequence. Considering previous studies that have highlighted the energetically favoured structures of C-rich triplex-forming oligonucleotide (TFO) sequences as those containing a higher number of non-sequential N3-protonated form [33], the cytosines in the PNA were considered in either the N3-imino (Ci) or the N3-protonated form (C+). Thus, two systems were designed: P3, with three C+ and four Ci, and P4, which featured four C+ and three Ci (Supplementary Table S1), showcasing different patterns of alternation between imino and protonated cytosines.

#### 2.6.2 Unbiased MD simulations

The optimization, equilibration, and production of MD simulations were performed using the Amber 18 suite [34]. The systems were parametrized using the leap module of Ambertools 18, applying the Amber force field (ff14SB for terminal lysine and ff99 + bsc0 for bcl2midG4-ds). For the PNA, Sanders et al. parameters were used [35], and the charge distribution for N3-protonated and N3-imino forms of cytosine was refined using RESP charges [36] fitted to HF/6-31G(d) electrostatic potential obtained with Gaussian 09 [37]. TIP3P water molecules were added to each system, ensuring a minimum spacing of 10 Å from the box edges, and the systems were filled in a truncated octahedron shape. Na^+^ counterions were added to neutralize each system. To optimize the geometry and eliminate bad contacts, each initial system underwent to six energy minimization steps, following the procedure described in Supplementary Table S2.

The steepest descent algorithm and positional restraints masks were used for the three initial steps with a force constant of 50 kcal/(mol Å^2^). The subsequent steps used the conjugate gradient algorithm with a lower force constant, applying restraints solely to the phosphodiester atoms of the DNA backbone. Distance restraints were imposed on the hydrogen bonds of complementary nucleotides and gradually relaxed over 5 ns to optimize the heterotriplex system. All systems were thermalized to 300 K, and the solvent box was further equilibrated for 1 ns. Then, three different schemes of equilibration were applied to the bcl2midG4-ds/TF-PNA1K heterotriplex systems.

i. The Hoogsteen (H) and Watson-Crick (WC) base-pairs restraints were gradually relaxed by decreasing the force constant over 4 ns of simulations (systems P3H and P4H) at 300 K, allowing the relaxation of TF-PNA1K around the restrained DNA double helix;
ii. Vice versa, the restraints were gradually decreased from the WC base pairs over 4 ns and later from the H base pairs (systems P3W and P4W), so allowing the relaxation of the DNA double helix against the restrained TF-PNA1K;
iii. Only H and WC H-Bond base pairs distance restraints were applied for 4 ns (systems P3HW and P4HW).

MD production was carried out in the isobaric-isothermal ensemble using a time step of 2 fs. Thermostat regulation was achieved through the Langevin dynamics approach with a collision frequency of 1.0 ps^−1^. The SHAKE algorithm was employed to constrain all hydrogen atoms, and periodic boundary conditions were implemented under constant pressure and temperature. The particle mesh Ewald method was utilized to handle long-range electrostatic interactions, with a cutoff of 9 Å applied for non-bonded interactions. The simulations were run using pmemd.cuda module implemented in Amber18 on NVIDIA GPUs. Two trajectories of 1 μs were conducted on the duplexes, while six trajectories of 1 μs were performed for the heterotriplexes, resulting in a total aggregate of 8 μs. The trajectories and related snapshots were inspected with Pymol [38] and VMD [39], while the post-processing analysis of trajectories was performed using Ambertools18 [34]. The different systems were classified into clusters using the CPPTRAJ module [40] and the Density-Based Spatial Clustering of Applications with Noise (DBSCAN) algorithm [41]. The analysis was performed on the combined trajectories of the three independent simulations (HW, H, W) for each P3 and P4 system. To determine the values for the minimum number of points to form a cluster (Minpoints) and the distance cutoff for creating a cluster (Epsilon, Ɛ), a *K*-distance plot was generated. The analysis revealed that both curves began to flatten out at approximately 1.8 and 1.4 Å for the P3 and P4 systems, respectively (Supplementary Fig. S4-cMD, panels A, B). The three independent trajectories, each ∼1000k frames long, corresponding to ∼1 μs, for a total of 3 μs of simulation, were compared. Metrics such as Davies-Bouldin Index (DBI) and the pseudo-F statistic (pSF), as well as the cluster size and cluster populations versus time, indicated favourable cluster quality (Supplementary Table S3 and Supplementary Fig. S4-cMD, panels A–D).

#### 2.6.3 Accelerated MD simulations

The final snapshot from the three equilibration steps of P3 and P4 systems (HW, H, W) was used as the initial structure for the dual-boost accelerated MD (aMD) applying a bias to the total potential energy function (*V*_*(r)*_) with an extra boost on the torsional term (*γ*). In aMD, transition events between distinct conformational minima are accelerated by adding a boost potential, *V*_*(r)*_, to the original potential energy, *V*_*(r)*_, only when *V*_*(r)*_ is below a chosen threshold boost energy (*Ep*). This leads to raise the potential surfaces near the minima while keeping those near the barriers or saddle points unchanged [42,43]. The aMD modification of the potential, *V*_(r)_*, is defined by the following equation (1) and 2:

(1) $${V_{\left(r\right)}}^{*}=V_{\left(r\right)}+{\Delta V}_{\left(r\right)}$$

(2) $${\Delta V}_{\left(r\right)}=\frac{{\left(Ep-V_{\left(r\right)}\right)}^{2}}{\left(\alpha P+Ep-V_{\left(r\right)}\right)}+\frac{{\left(Ed-{Vd}_{\left(r\right)}\right)}^{2}}{\left(\alpha \gamma +Ed-{Vd}_{\left(r\right)}\right)}$$

where *V(r)* and *Vd(r)* are the normal energy potential and normal torsion potential, respectively; *Ep* and *Ed* are user-defined thresholds respectively based on the average potential and dihedral energy obtained from the equilibration of each system. The terms *αP* and *αγ* are the acceleration parameters that determine the shape of the modified potentials [[42], [43], [44]]. For our aMD simulations, we used a specific set of acceleration parameters (*E* and *α*) as shown in Supplementary Table S4. A production trajectory of 500 ns was generated for each system. These trajectories were then clustered based on the K-distance plots for the DBSCAN tuning parameter, considering values at around 2.8 and 3.0 Å for P3 and P4 systems, respectively (Supplementary Table S3 and Supplementary Fig. S4-aMD, panels A–D).

#### 2.6.4 Principal component analysis

Principal component analysis (PCA) was performed using the CPPTRAJ module [40] to compare the essential motions in both cMD and aMD simulations. The analysis was executed on the heavy atoms of the systems (residues from 1 to 30), excluding the C-terminal NH_2_CO-Lys. The trajectories of each equivalent system were merged to create a structure, which served as the reference for the root-mean-square (RMS) fitting to eliminate global translational and rotational motions. The covariance matrix was then calculated on the Cartesian coordinates of the trajectories, and the matrix was diagonalized to obtain the eigenvectors (principal components, PCs). The first two PCs projections (PC1, PC2), representing the highest variance of motions, were projected and plotted as scatter plots of the free energy landscape. The dominant states were statistically evaluated assuming a Boltzmann-weighted distribution of the PCs, providing insights into the conformational dynamics of the systems.

### 2.7 Ad5Delta24 oncolytic adenovirus (OAd) preparation and complexation with TF-PNA6K

The OAd was generated, amplified, and characterized as reported elsewhere [45,46]. OAd-TF-PNA6K complexes were formed by combining OAd and TF-PNA6K at a ratio of 1:100 (w/w) following the specified protocol: (i) the corresponding amount of micrograms of viral protein was calculated for each microliter of the viral preparation used; (ii) 100 μg of TF-PNA6K was added for each microgram of viral protein, with the volume adjusted to 100 μL using sterile Milli-Q water (VWR International Srl, Milano, Italy); (iii) after vortexing, the mixtures were incubated for 15 min at room temperature; and (iv) vortexed again just before use. Fresh reagents were used to prepare new OAd-TF-PNA6K complexes for each experiment.

### 2.8 Zeta potential and dynamic light scattering (DLS) analysis

Zeta potential measurements were performed at 25 °C using a Malvern Zetasizer Nano ZS System (Malvern Panalytical, Worcestershire, UK). Before measurements, OAd-TF-PNA6K complexes were subjected to vortexing and subsequent dilution with sterile Milli-Q water at the final volume of 700 μL, adjusting the pH to 7.4, before being transferred into DTS1060 disposable capillary cells (Malvern Panalytical, Worcestershire, UK). We used the same disposable capillary cells for the DLS analysis, selecting different parameters on the Malvern Zetasizer Nano 3.30 software. Five measurements were performed for Zeta potential and DLS analysis.

### 2.9 Cell culture and viability assay

Human A549 lung adenocarcinoma and MDA-MB-436 triple negative breast adenocarcinoma cell lines were purchased from the American Type Culture Collection (ATCC; Manassas, VA, USA). The cells were grown in adhesion at 37 °C in a 5 % CO_2_ humidified atmosphere using the Dulbecco's modified Eagle's medium (DMEM) (Gibco, Waltham, MA, USA) supplemented with 10 % heat-inactivated fetal bovine serum (FBS, Gibco), penicillin (50 U/mL), streptomycin (500 μg/mL), and glutamine (4 mmol/L). To assess cell viability, the cells were seeded in 96-well plates at the density of 2.5 × 10^3^ cells/well. 24 h after seeding, the cells were treated with 100 μL of OAd or OAd-TF-PNA6K preparations corresponding to 0.1, 1, 10, and 100 viral particle (VP)/cell doses in cell culture medium for 1, 3, or 5 days. Each OAd-TF-PNA6K preparation was obtained at the 1:100 OAd/TF-PNA6K ratio (w/w). The viability of cells treated with the TF-PNA6K alone was assessed by using TF-PNA6K concentrations corresponding to those used for the OAd-TF-PNA6K preparations (serial dilutions of the 0.33 nM solution corresponding to the amount of TF-PNA6K used to prepare the 100 VP/cell dose). The MTS assay was performed at each time point according to the manufacturer's protocol (CellTiter 96 Non-Radioactive Cell Proliferation Assay; Promega, Nacka, Sweden). A PerkinElmer Multimode microplate Reader (Waltham, MA, USA) was used to acquire the spectrophotometric data. Cytotoxicity was evaluated in triplicate and was expressed as the percentage of viable treated cells compared to untreated cells.

### 2.10 Statistical analyses and correlation models

Statistical analyses were conducted utilizing the GraphPad Prism 6 software (GraphPad Software, Inc., La Jolla, CA, USA). The data were presented as mean ± standard deviation (SD). For the comparison of repeated results, a two-way analysis of variance (ANOVA) was employed, and statistical significance was considered for P-values <0.05.

## 3 Results

### 3.1 Assessment of the interaction between TF-PNA1K and the bcl2midG4 duplex and quadruplex targets

**Fig. 1:**
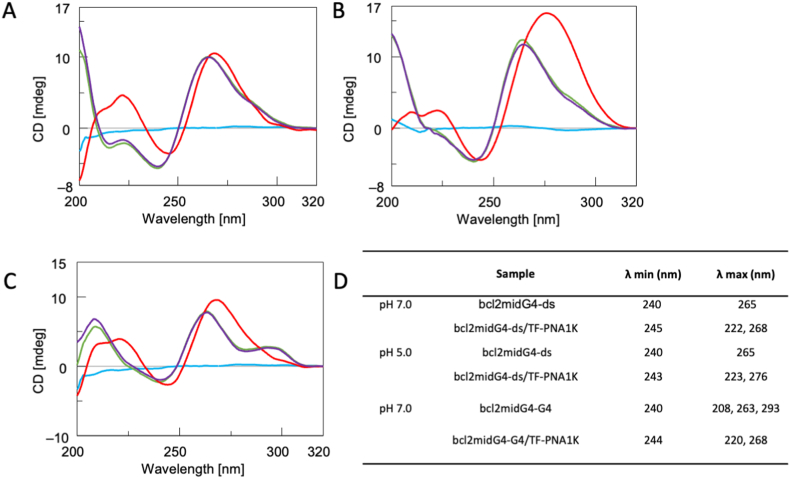
CD spectra of bcl2midG4-ds before (purple line) and after (red line) incubation with 5 equivalents of TF-PNA1K at (A) pH 7.0 or (B) pH 5.0. (C) CD spectra of bcl2midG4-G4 before (purple line) and after (red line) incubation with the same excess of TF-PNA1K at pH 7.0. All samples were dissolved in a 60 mmol/L K^+^-containing buffer. The CD profiles of the arithmetic sum of DNA + TF-PNA1K and TF-PNA1K alone are reported as green and light blue lines, respectively. All spectra were acquired at 5 °C and normalized at 320 nm. (D) Table with λ values for CD minima and maxima of each sample at pH 7.0 or 5.0. (For interpretation of the references to colour in this figure legend, the reader is referred to the Web version of this article.)

To assess the interaction and the binding mode of TF-PNA1K with the bcl2midG4-ds and bcl2midG4-G4 targets, we performed CD, CD-melting, and PAGE studies. We first recorded the CD behaviour of the bcl2midG4 duplex before and after 24 h incubation with 5 equivalents of TF-PNA1K (Fig. 1). The study was performed at pH 7.0 (Fig. 1, panel A) and 5.0 (Fig. 1, panel B), in agreement with literature data that forecast enhanced stability for triplexes at acidic pH [26]. The spectra of bcl2midG4-ds alone are characterized by one negative band at 240 nm, one positive band at 265 nm, and a weak hump around 290 nm at both the studied pHs (Fig. 1A and B, purple line). On the other hand, the CD spectrum of the mixture obtained incubating TF-PNA1K with bcl2midG4-ds at pH 7.0 showed one negative band at 245 nm and two positive bands at 222 and 268 nm (Fig. 1A, red line). The spectrum of the same mixture incubated at pH 5.0 revealed one negative band at 243 nm and two positive bands at 223 and 276 nm (Fig. 1B, red line), with a significant enhancement of the positive dichroic signal at 276 nm. Overall, the analysis of the CD spectra of the bcl2midG4-ds/TF-PNA1K mixtures confirmed the interaction of TF-PNA1K with the bcl2midG4-ds under both the studied pH conditions, with the observed CD profiles being in close agreement with those previously reported for DNA-PNA heterotriplexes [27]. Subsequently, we recorded the CD profile of the bcl2midG4 G-quadruplex, both before and 24 h after incubation with 5 equivalents of TF-PNA1K (Fig. 1C, purple and red lines, respectively). This analysis aimed to assess whether TF-PNA1K retained the capability, similar to its shorter analogue, to bind the G4 structure formed by the bcl2midG4 G-rich DNA sequence (bcl2midG4_G). The bcl2midG4_G is well-known for folding into a 3 + 1 hybrid-type G-quadruplex (bcl2midG4-G4) with a CD spectrum characterized by two positive bands centered at 263 and 293 nm, along with one negative band centered at 240 nm [47]. Upon incubation with TF-PNA1K, the CD profile of bcl2midG4-G4 exhibited changes. Specifically, the positive CD hump at 293 nm disappeared, and the remaining positive and negative signals showed a red shift of approximately 5 nm. Notably, the CD spectrum of the 1:5 bcl2midG4-G4/TF-PNA1K mixture differed significantly from the simple arithmetic sum of the individual components (Fig. 1C, green line). This observation confirmed that TF-PNA1K, alike its shorter 7-mer analogue, effectively binds to the G-quadruplex-folded bcl2midG4_G sequence, forming a new complex with a CD signature almost superimposable to that of the bcl2midG4-ds/TF-PNA1K heterotriplex complex. The λ values of CD minima and maxima recorded for each sample at the two studied pHs are summarized in Fig. 1D.

**Fig. 2:**
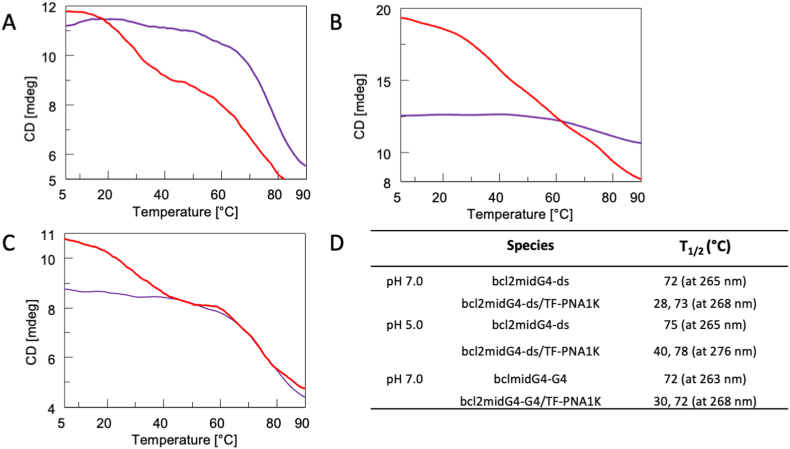
CD melting curves of: A) bcl2midG4-ds in potassium buffer at pH 7.0 before (purple line) and after (red line) the addition of TF-PNA1K at the 1:5 DNA/PNA ratio; B) bcl2midG4-ds in potassium buffer at pH 5.0 before (purple line) and after (red line) the addition of TF-PNA1K at the 1:5 DNA/PNA ratio; C) bcl2midG4-G4 in potassium buffer at pH 7.0 before (purple line) and after (red line) the addition of TF-PNA1K at the 1:5 DNA/PNA ratio; D) Calculated T_1/2_ (°C) of each sample. (For interpretation of the references to colour in this figure legend, the reader is referred to the Web version of this article.)

To assess the thermal stability of bcl2midG4 duplex and quadruplex secondary structures before and after their incubation with TF-PNA1K, we performed a CD melting study on the samples annealed in 60 mmol/L K^+^ buffer at pH 7.0 or 5.0, whose results are summarized in Fig. 2D. The CD melting curve of bcl2midG4-ds/TF-PNA1K at pH 7.0 (Fig. 2A, red line) showed two inflections at 28 and 73 °C. The first was attributable to the dissociation of the TF-PNA1K strand from the DNA_2_-PNA heterotriplex, while the second to the melting of the bcl2midG4-ds duplex (Fig. 2A, purple line). We observed two less-resolved transitions at 40 and 78 °C in the melting curve of the bcl2midG4-ds/TF-PNA1K sample incubated at pH 5.0 (Fig. 2B, red line). The observed 12 °C enhancement in the apparent dissociation temperature of bcl2midG4-ds/TF-PNA1K triplex was in agreement with the expected higher stability of triplexes at acidic pH [26]. The CD melting curve of bcl2midG4-G4/TF-PNA1K also showed the presence of two clear inflection points (Fig. 2C, red line), the first at 30 °C and the second at 72 °C. Considering that the calculated melting temperature for the 10 bp long 5′TGGTTGGGGG3′/5′CCCCCTTCCT3′ DNA duplex in 60 mM monovalent cation is 39.8 °C (determined using the Oligo Analyzer 3.1 web tool at https://eu.idtdna.com/calc/analyzer) and that the thermal stability of DNA-PNA heteroduplexes is higher than that of the corresponding natural counterpart [1], we attribute the first inflection point to the disruption of the bcl2midG4_G/(TF-PNA1K)_2_ heterotriplex complex, in agreement with the CD evidence, and the second inflection point to the melting of the bcl2midG4 G-quadruplex which readily forms after the dissociation of the two PNA strands from the G-rich bcl2midG4_G DNA strand.

**Fig. 3:**
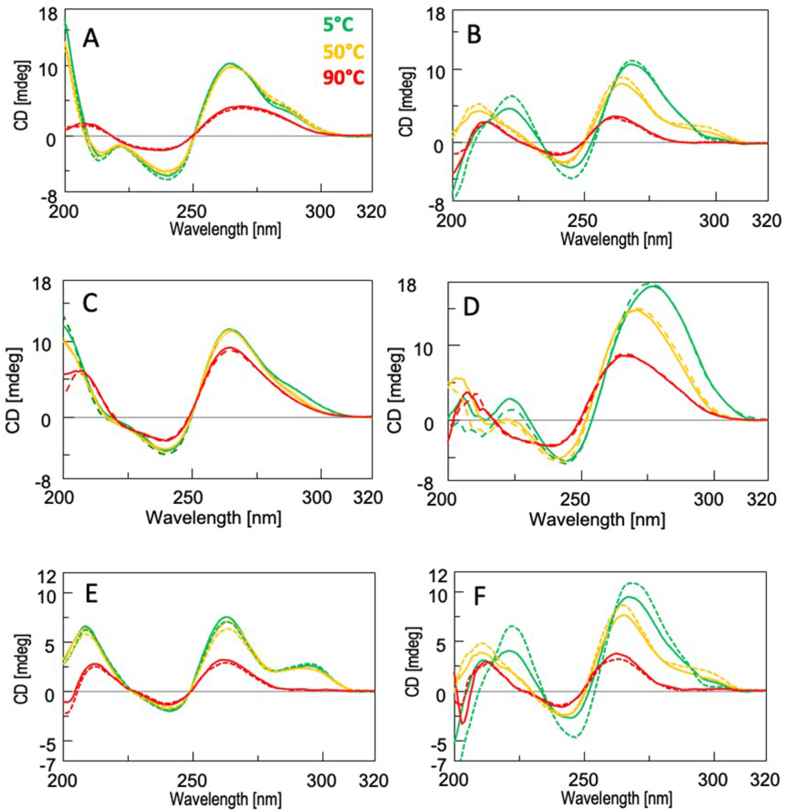
CD profiles recorded in 60 mmol/L potassium buffer for bcl2midG4-ds alone at pH 7.0 (A) and 5.0 (C), bcl2midG4-ds/TF-PNA1K (at a 1:5 equivalents ratio) at pH 7.0 (B) and 5.0 (D), bcl2midG4-G4 alone at pH 7.0 (E), bcl2midG4-G4/TF-PNA1K (at a 1:5 equivalents ratio) at pH 7.0 (F) at 5 °C (green), 50 °C (yellow), and 90 °C (red). Heating: solid line; cooling: dashed line. (For interpretation of the references to colour in this figure legend, the reader is referred to the Web version of this article.)

We also recorded the CD spectra at 5, 50, and 90 °C during the heating (solid curves) and cooling (dashed curves) runs of the CD thermal analyses (Fig. 3). In the absence of the PNA ligand, the CD spectra of bcl2midG4-ds recorded before the thermal denaturation and after the cooling were almost superimposable, both at pH 7.0 and pH 5.0 (Fig. 3A and C, respectively), and the same was observed for the corresponding CD spectra of bcl2midG4-G4 at pH 7.0 (Fig. 3E). These data confirmed that the studied bcl2midG4 DNA samples consistently fold into a single well-defined secondary structure when annealed alone in the 60 mmol/L K^+^-containing buffer. Different behaviours were observed when the two pre-folded secondary structures were incubated in the presence of TF-PNA1K. In the case of the bcl2midG4-ds/TF-PNA1K samples, apart from the major hyperchromic and bathochromic effects on the positive dichroic signal at 265 nm caused by the pH decrease from 7.0 to 5.0, we did not observe any significant difference between the CD spectra recorded during the heating and cooling steps at both pHs and at each sampled temperature (Fig. 3B and D, respectively). Conversely, the two CD spectra recorded at 5 °C for the bcl2midG4-G4/TF-PNA1K system (green curves in Fig. 3F) differed significantly from the spectra obtained for bcl2midG4-G4 alone at the same temperature (green curves in Fig. 3E). The former were characterized by the absence of the hump at 293 nm and by a significant enhancement in the positive dichroic signal centered at 265 nm for the spectrum recorded at the end of the cooling run compared to the spectrum recorded before the melting run at the same temperature (Fig. 3F). Taken together, this evidence confirmed the ability of the new TF-PNA1K to bind the bcl2midG4 G-quadruplex and suggested that more than one binding mode is possible, as disclosed by the comparison of the different CD spectra obtained at the beginning and the end of the CD melting experiment. The CD spectra of bcl2midG4-G4/TF-PNA1K recorded at 50 °C during the heating and cooling processes were characterized by the presence of the hump at around 293 nm, consistent with the presence of the unliganded bcl2midG4-G4 quadruplex at that temperature (Fig. 3F, yellow lines). Indeed, the spectrum recorded at 50 °C during the cooling run was almost superimposable to that of bcl2midG4-G4 in the absence of PNA recorded at the same temperature (Fig. 3E, yellow dashed line), thus indicating the absence of any significant interaction between the G4 and the TF-PNA1K at 50 °C. This latter finding also suggests that TF-PNA1K interacts more rapidly with the bcl2midG4 double helix than with the G4 and that the former interaction results in the formation of a single heterotriplex structure.

**Fig. 4:**
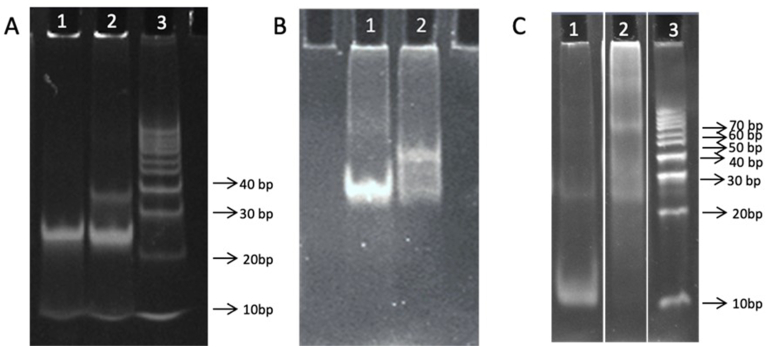
PAGE of bcl2midG4-ds (A, B) and bcl2midG4-G4 (C) alone or incubated with TF-PNA1K in potassium solution at pH 7.0 (A and C) or pH 5.0 (B). Lanes 1A and 1B, bcl2midG4-ds; lanes 2A and 2B, bcl2midG4-ds incubated with TF-PNA1K. Lane 1C, bcl2midG4-G4; lane 2C, bcl2midG4-G4 incubated with TF-PNA1K. Lanes 3A and 3C, DNA duplex ladder (10−100 bp).

The molecular size and the DNA/PNA stoichiometry of the complexes formed after incubating bcl2midG4-ds and bcl2midG4-G4 with TF-PNA1K were examined by nondenaturing polyacrylamide gel electrophoresis (Fig. 4A–C and Supplementary Fig. S5). As expected, the 23 bp-long bcl2midG4 duplex migrated almost exclusively as a single band with an R_F_ in between the 20 and 30 bp reference DNA ladder (Fig. 4A, lane 3), both at pH 7.0 (Fig. 4A, lane 1) and 5.0 (Fig. 4B, lane 1). After the addition of 5 equivalents of TF-PNA1K, we noticed the appearance of a new slower band associable with the formation of a heterotriplex complex. At pH 7.0, the intensity of the band attributable to the triplex complex was lower than that assigned to the bcl2midG4 duplex (Fig. 4A, lane 2), whereas at pH 5.0 we observed the opposite (Fig. 4B, lane 2), in agreement with the above-reported CD results and literature data regarding the higher amount of triplex formed at acidic pH [26]. As previously reported, the PAGE of bcl2midG4-G4 gave two principal bands corresponding to the monomeric and dimeric G-quadruplexes [18] (Fig. 4C, lane 1). After the incubation with 5 equivalents of TF-PNA1K, we saw the disappearance of the band corresponding to the bcl2midG4-G4 monomeric G-quadruplex and the appearance of new intense slower bands attributable to the formation of multimeric complexes (Fig. 4C, lane 2).

### 3.2 Characterization of bcl2midG4-ds/TF-PNA1K heterotriplex structure by conventional (cMD) and accelerated (aMD) molecular dynamics simulations

**Fig. 5:**
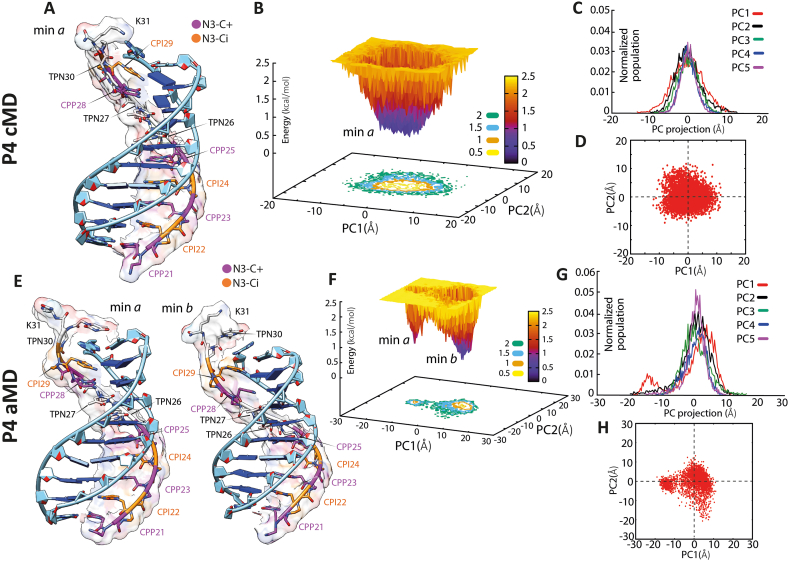
P4 System. Representative structure of the energetic minima and free energy surface (FES) and principal component (PC) plots of the cMD (A and B–D, respectively) and aMD (E and F–H, respectively) simulations. In B and F, density-coloured landscapes represent the FES distributions: blue areas denote higher density, while yellow ones denote low density. On the right in the upper, the PC projection histograms of cMD (C) and aMD (G) simulations, calculated on the phosphate (P) and nitrogen (N4) atoms of DNA and PNA respectively, excluding C-terminal NH_2_CO-Lys; bottom: the first two PCs calculated on the aggregate of cMD (D) and aMD (H) trajectories for each system. The heterotriplex structures were extracted by filtering the values of the PCs. DNA and PNA backbones are shown in cartoons, while sugar and bases are represented in sticks and bricks. The carbon atoms of the N3-protonated cytosines (N3–C+) and the N3-imino form (N3-Ci) are highlighted and labelled magenta and orange. (For interpretation of the references to colour in this figure legend, the reader is referred to the Web version of this article.)

**Fig. 6:**
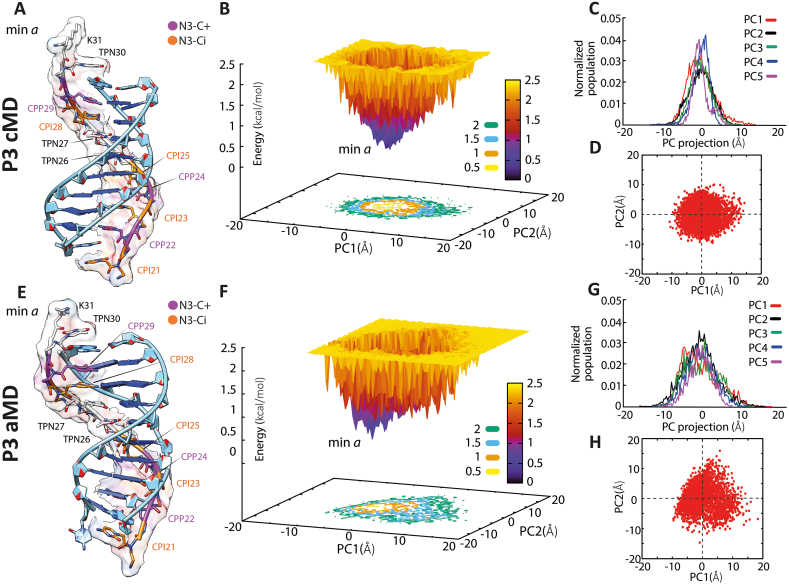
P3 System. Representative structure of the energetic minima and free energy surface (FES) and principal component (PC) plots of the cMD (A and B–D, respectively) and aMD (E and F–H, respectively) simulations. In B and F, density-coloured landscapes represent the FES distributions: blue areas denote higher density, while yellow ones denote low density. On the right in the upper, the PC projection histograms of cMD (C) and aMD (G) simulations, calculated on the phosphate (P) and nitrogen (N4) atoms of DNA and PNA respectively, excluding C-terminal NH_2_CO-Lys; bottom: the first 2 PCs calculated on the aggregate of cMD (D) and aMD (H) trajectories for each system. The heterotriplex structures were extracted by filtering the values of the PCs. DNA and PNA backbones are shown in cartoons, while sugar and bases are represented in sticks and bricks. The carbon atoms of the N3-protonated cytosines (N3–C+) and the N3-imino form (N3-Ci) are highlighted and labelled magenta and orange. (For interpretation of the references to colour in this figure legend, the reader is referred to the Web version of this article.)

To assess the stability and the structural properties of the heterotriplex consisting of TF-PNA1K and the ten nucleotides-long truncation of the bcl2midG4-ds duplex (referred to as bcl2midG4-ds10), we used molecular dynamics simulations. The conformational behaviour of the bcl2midG4-ds10 was initially explored through two cMD simulations, starting from the canonical A-form and B-form of the duplex. Both trajectories converged to a dominant conformational state, which accounted for 82.4 % of the total population according to the clustering analysis (Supplementary Table S5). The analysis of helicoidal parameters (Supplementary Table S5) of the dominant conformer exhibited hybrid characteristics, displaying features of both A-type (negative slide and X-displacement) and B-type (Zp) DNA structures. Therefore, the major groove of bcl2midG4-ds10 widened compared to canonical A- and B-DNA structures. Interestingly, the cMD-derived structure of bcl2midG4-ds10 closely resembled the duplex in an NMR model of a Py-Pu-Py triplex (PDB ID: 149D [32]; RMSD 1.3 Å). Thus, this cMD-derived structure was utilized as the initial duplex structure for building the heterotriplex system with TF-PNA1K. The study investigated two different PNA molecular species, P3 and P4, which differed in the number and position of N3-protonated cytosines (Supplementary Table S1). Previous studies on the effect of consecutive cytosines protonation on triplex stability were considered [26,33], and it was assumed that the remaining cytosines in the PNA triplexes were in the N3-imino form, as reported for d (G•C•C) triplexes [33]. Due to the lack of experimental structures as a template for bcl2midG4-ds10/TF-PNA1K heterotriplex, the systems were manually constructed following the procedures described in the experimental section and equilibrated following three different strategies. Initially, three independent cMD simulations of 1 μs each were conducted for a total simulation time of 3 μs for both P3 and P4 systems (H, W, HW). Analysis of the RMSD plots over time (Supplementary Fig. S6) and cluster analysis (Supplementary Fig. S4; Supplementary Table S3) indicated stable and convergent trajectories. To explore the conformational space further, 500 ns of accelerated MD (aMD) simulations were performed for P4 and P3 systems, allowing enhanced conformational sampling beyond the reach of conventional MD [48]. Visual inspection of the simulations and the analysis of RMSD over time (Supplementary Fig. S6 panels A and B, respectively) demonstrated stable simulations with slightly lower average RMSD for the P4 system, which had a greater number of N3-protonated cytosines (N3–C+), compared to the P3 system. Free energy surface (FES) and principal component (PCs) plots were generated for both cMD (panels B–D) and aMD (panels F–H) simulations of the P4 and P3 systems (Fig. 5, Fig. 6, respectively) to compare the sampled conformational space and to identify representative energy minima. In the cMD simulation of P4, a minimum was found characterized by the absence of a H-bond interaction between TPN30 and A11 at the C-terminus, while all WC base pairs were preserved (Fig. 5, panels A and B). The aMD enhanced method explored additional conformational space and identified two metastable minima (min *a*, min *b*) with an energy difference of less than 1.5 kcal/mol (Fig. 5, panels E and F). The structure in min *b* closely resembled the minimum found in the cMD simulations, while the higher energy minimum (min *a*) differed from min *b* by the loss of a WC base pair interaction (A11:T10). Accordingly, the H-bond occupancy plot (Supplementary Fig. S7) confirmed these findings, showing the loss of Hoogsteen base pair A11:TPN30 and a slight weakening of the WC base pair A11:T10 during the cMD simulation. In the aMD simulation, both the H and WC base pairs exhibited more pronounced weakening.

The analysis of the P3 simulations revealed a slightly less stable complex due to the lower number of protonated cytosines. Free energy surface (FES) and principal component (PC) plots obtained from cMD and aMD simulations on the P3 system showed only one energetic minimum. However, these minima were characterized by the loss of H base pairs at both ends (TPN30:A11 and CPI21:G20) and the WC base-pair G20:C1 (Fig. 6). The analysis of H-bond occupancy confirmed the complete loss of terminal Hoogsteen pairs (A11:TPN30 and G20:CPI21) in both cMD and aMD and weakening of the WC terminal base pairs (Supplementary Fig. S7). Additionally, the stability of the WC base pairs exhibited similar behaviour in both complexes.

It should be underlined that the simulated systems were limited to the complementary region of bcl2midG4-ds/TF-PNA1K triplex complex. Therefore, the frying effect observed at both ends of the duplex structures in the four analysed complexes would not be present in the biological system. The H-bond occupancy plot (Supplementary Fig. S7) showed that the loss of H base pairs is only partially related to the extent of WC base pair, rather it is dependent by the chemical nature of bases involved. The A11:TPN30 and G20:CPI21 showed a negligible occupancy in all the system studied, despite the occupancy of the corresponding WC base pair. On the contrary, G20:CPP21 Hoogsteen interaction resulted stable even in the terminal position. These combined analyses provided insights into the dynamic behaviour and stability of the analysed triplex system, indicating that the protonation pattern of the P4 system significantly influenced triplex stability but did not affect duplex stability. The aMD simulations highlighted lower stability of the Hoogsteen base pairs formed by the imino cytosines (CPI) compared to the protonated (CPP) form. Overall, these findings align with previous studies on DNA triplexes, where optimal stability with C-rich TFOs was observed with a greater number of non-subsequent N3-protonated cytosines (29). Finally, the helix parameters of the bcl2midG4-ds10/TF-PNA1K triplex were analysed in the representative clusters identified for P3 and P4 systems and compared with parameters obtained for the main cluster of the bcl2midG4-ds10 alone and the averaged NMR ensemble of a Py-Pu-Py triplex (PDB ID: 149D) (Supplementary Table S5). The P3 and P4 triplexes showed the same overall structure, indicating that the protonation state does not significantly affect the curvature of the structure. Comparisons with the helicoidal structure of the duplex suggested that the interaction with the TF-PNA slightly influenced the helicoidal parameters, except for the major groove, which was wider in the triplex structure compared to the duplex. Comparisons with the helicoidal structure of a DNA triplex (PDB ID: 149D) revealed minor differences in roll, helical twist, and major groove, indicating that the PNA-containing triplex differed from DNA triplexes by having a wider major groove and slight unwinding of the target structure, consistent with previous findings on DNA-PNA heteroduplexes [13].

### 3.3 Formation of the oncolytic adenovirus OAd-TF-PNA6K complex

**Fig. 7:**
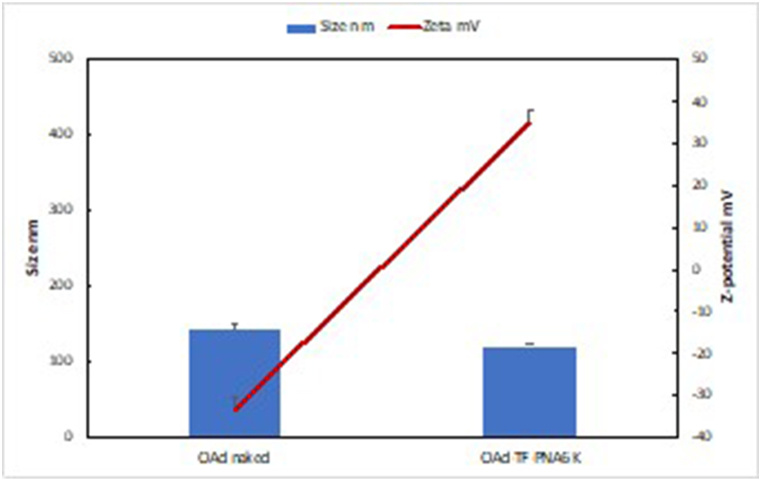
Hydrodynamic diameter (nm) and zeta potential (mV) values of OAd-TF-PNA6K complexes at the 1:100 virus to PNA ratio.

OAds have been exploited for their capability to recognize and selectively lyse tumour cells over healthy tissues and to transport cargos adsorbed on their surface within cancer cells. Recently, we demonstrated that positively charged PNAs could be efficiently delivered in cancer cells when transported on the negatively-charged surface of OAds [18]. Herein, we wanted to assess the anti-Bcl-2 efficacy of the newly designed triplex-forming PNA (TF-PNA6K) – bearing an extended positively charged lysine tail to enhance the electrostatic interaction with the OAd surface – complexed or not with Ad5Delta24 oncolytic adenoviruses. To this end, we first studied the formation of the complex using the 1:100 OAd:TF-PNA6K ratio and monitoring the charge and the size of the resulting systems by using zeta potential and dynamic light scattering (DLS) measurements, respectively (Fig. 7). The net charge of the OAd raised from −33.16 to +34.76, respectively for the bare OAd and OAd-TF-PNA6K complex. At the same time, the corresponding hydrodynamic diameter decreased from 143.56 to 121.28 nm. These results confirmed that the coating with the positively charged TF-PNA6K reverses the charge of OAds not affecting the colloidal stability, as evidenced by the polydispersion index (PDI = 0.140 for OAd and 0.122 for OAd-TF-PNA6K). In agreement with previous zeta potential and DLS evidence, we confirmed the 1:100 OAd-TF-PNA6K ratio as the most suitable for the successive cell viability studies.

### 3.4 Evaluation of cell viability on A549 and MDA-MB-436 cancer cell lines

**Fig. 8:**
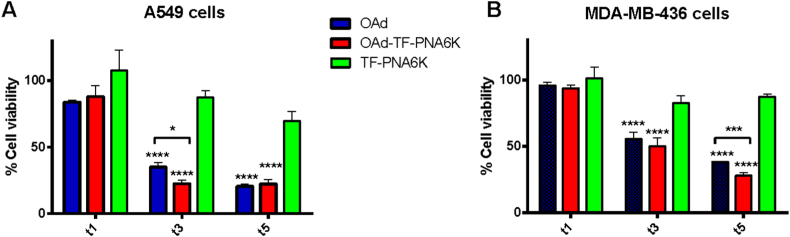
Viability of A549 (A) and MDA-MB 436 (B) cells after one, three, and five days (indicated as t1, t3, and t5) of treatment with OAd (100 VP/cell), OAd-TF-PNA6K (100 VP/cell) or TF-PNA6K alone. Cell viability is reported as the percentage of viable cells compared to untreated cells. Significance was assessed using the two-way ANOVA, *p < 0.05, ***p < 0.001, ****p < 0.0005.

Moreover, we decided to evaluate the biological effect of the optimized OAd-TF-PNA6K complex on cellular models. To this scope, using the 3-(4,5-dimethylthiazol-2-yl)-5-(3-carboxymethoxyphenyl)-2-(4-sulfophenyl)-2H-tetrazolium (MTS) assay we analysed the cell viability of two different human cell lines known to be permissive for human oncolytic adenoviruses and expressing high levels of the Bcl-2 protein, i.e., the lung adenocarcinoma A549 (Fig. 8A) and the triple-negative breast adenocarcinoma MDA-MB-436 (Fig. 8B). The MTS assay was carried out after one, three, and five days of incubation with either 0.1, 1, 10, and 100 viral particles (VP)/cell + 1:100 (w/w) OAd/TF-PNA6K preparations or with the negative control (OAd alone) at the same doses. For each time point, we also measured the cell viability after treatment with TF-PNA6K alone at concentrations corresponding to those used for the TF-PNA6K-coated OAd preparations (Supplementary Fig. S8). The viability of treated cells was expressed as a relative percentage compared to untreated control cells. Treatment with TF-PNA6K alone caused only a modest reduction of cell viability after three or five days of incubation at all tested concentrations in both cell lines compared to untreated controls (Fig. 8 and Supplementary Fig. S8). Cells treated with OAd alone showed a significant oncolytic effect on day 3 at all concentrations in both cell lines (viability 35–42 % for A549 and 56–68 % for MDA-MB-436 cells). At the same time point, the viability of A549 and MDA-MB 436 cells treated with OAd-TF-PNA6K was quite similar to that observed for cells treated with naked OAd at all tested concentrations, except for A549 cells incubated with 100 VP/cell for which the cell viability due to treatment with OAd-TF-PNA6K (23 %) was statistically significantly lower (p < 0.05) than that induced by treatment with naked OAd (35 %) (Fig. 8). The MTS readings performed on day 5 from the incubation revealed a different outcome for the two studied cancer cell lines because of their different permissivity to the Ad5Delta24 oncolytic viral strain. For the more permissive A549 cells, on day 5 it was impossible to appreciate any cytotoxicity enhancement due to the viral coating with TF-PNA6K because of the advanced progression of the virus-induced oncolysis (viability 21 % and 22 %, respectively, for cells treated with OAd or OAd-TF-PNA6K). On the contrary, for the less permissive MDA-MB-436 cells, the cytotoxicity of TF-PNA6K-coated OAds recorded on day 5 from the incubation (viability 28–42 %) was higher than that induced by the naked OAd (38–46 %) at all viral doses. The enhancement of viral cytotoxicity induced by the coating with TF-PNA6K was statistically significant at 10 and 100 VP/cell doses (Supplementary Fig. S8).

## 4 Discussion

In a previous paper [18] we demonstrated that the 7-mer PNA sequence complementary to the longest loop of the G-quadruplex structure formed by the bcl2midG4 DNA sequence located in the P1 promoter of the Bcl-2 proto-oncogene was capable of downregulating the expression of Bcl-2 protein in A549 and MDA-MB-436 adenocarcinoma cells when complexed with an oncolytic adenovirus used as the PNA vector, thus exerting a synergistic anticancer outcome. Considering the low statistical representativeness of the G-quadruplex-folded bcl2midG4_G promoter sequence and the sub-optimal sequence specificity of the 7-mer PNA, we decided to elongate the PNA sequence by adding three cytosines (complementary to the G_17-19_ segment of the target bcl2midG4 promoter sequence) at its N-terminal (Supplementary Fig. S9) to enhance the binding specificity and affinity of the resulting TF-PNA probe for the Bcl-2 P1 promoter. Differently from its shorter analogue, TF-PNA1K, bearing one lysine residue at its C-end to allow water solubility, can bind the complementary bcl2midG4_G sequence both when it is folded in G-quadruplex (bcl2midG4-G4) or coupled with the complementary C-rich strand to form the corresponding duplex (bcl2midG4-ds). In the former case, TF-PNA1K induces the unfolding of the G-quadruplex and the formation of multimeric DNA-PNA complexes likely including the DNA/(PNA)_2_ heterotriplex, while in the latter it induces the formation of the bcl2midG4-ds/TF-PNA1K heterotriplex ((DNA)_2_/PNA). These complexes, being not recognized by DNA helicases, are expected to inhibit the binding of transcriptional factors on the Bcl-2 promoter with consequent anticancer effects. Differently from what observed for the interaction with the bcl2midG4 G-quadruplex, the addition of TF-PNA1K to the pre-folded bcl2midG4-ds at pH 7.0 did not induce significant variations in the CD spectrum of the DNA duplex, except for 3 and 5 nm red-shifts of the positive band at 265 nm and the negative band at 240 nm, respectively. Nonetheless, the binding of TF-PNA1K to the bcl2midG4 duplex was confirmed by the CD melting study that showed two sequential transitions at 28 and 73 °C ascribable, respectively, to the dissociation of the PNA from the heterotriplex and to the opening of the DNA duplex. Similarly, the addition of TF-PNA1K to the bcl2midG4-ds at pH 5.0 resulted in 11 and 3 nm redshift of the above-cited positive and negative CD bands, respectively, which was paired to a 12 °C enhancement in the melting temperature of the first inflection point of the CD melting curve (Fig. 2B). As for the latter observation, the protonation of cytosines in N3 positions of the TF-PNA1K, clearly favoured under the acid conditions mimicking the tumour microenvironment, increases Hoogsteen base-pairing with bcl2midG4-ds [27] thus being responsible for the 12 °C enhancement of the melting temperature value corresponding to the dissociation of the PNA strand from the triplex complex. PAGE studies suggested the formation of a (DNA)_2_/PNA heterotriplex complex that was favoured at pH 5.0 with respect to pH 7.0, as expected for the higher protonation degree of cytosines. Accordingly, MD simulations showed increased stability for the systems having a larger number of protonated cytosines (P4 vs. P3), but also suggested that an important role could be played by the position of the protonation sites in the TFO strand and by the PNA sequence itself, thus furnishing useful hints for the future design of TF-PNAs targeting G-rich DNA sequences. Structurally, MD simulations showed that the target C-rich DNA sequence folded in a hybrid non-A- and non-B-DNA helix. Interestingly, the helix is characterized by a wider major groove with respect to the canonical DNA structures, comparable to the width of the duplex in NMR triplex structures, thus suggesting that C-rich sequences may represent a suitable target for triplex formation. Interestingly, the interaction with the TF-PNA significantly affects the major groove of the DNA duplex embedded in the heterotriplex structure, which was shown to be wider with respect to the DNA duplex but also when compared to the DNA triplex, as revealed by MD using the reference homotriplex structure with PDB ID: 149D. Overall, MD simulations of bcl2midG4-ds/TF-PNA1K triplex offered a clear view of the dynamic behaviour and stability of the base-pair interactions in the (DNA)_2_/PNA heterotriplex structure. We observed in all the most representative structures the loss of the Hoogsteen interactions at the TPN30 site at the C-terminus of the PNA, as well as the loss of the Hoogsteen interactions at the N-terminus only in the P3 system, bearing the N3-imino base CPN21. To develop a more powerful delivery system for the PNA herein investigated, we complexed this positively charged oligonucleotide analogue with a naturally negatively-charged oncolytic adenovirus. This technique has been widely exploited to boost the efficacy of oncolytic viruses and to enhance the delivery of small peptides. For example, it has been shown an effective way to deliver tumour-specific peptides aimed at triggering specific immune responses [[49], [50], [51], [52]], pro-apoptotic peptides [53] and also PNAs [18]. First, we had to ascertain that a PNA with a sequence longer than those previously investigated could still be delivered by an oncolytic virus. Second, we were interested in evaluating whether this approach could enhance the efficacy of the virus alone. Satisfactorily, our results showed an increased efficacy in terms of cancer cell killing activity, as we proved using two different human cancer cell lines (A549 and MDA-MB-436), suggesting not only that oncolytic viruses might be used as a fast carrier for these molecules to the cells but also that a significant synergy between the PNA and the oncolytic virus occurs. We observed, though, significant differences between the two cancer cell models at the two different time points, which ultimately reflects the fact that cell infection, as well as virus replication, differ in the two studied cancer cell lines.

In summary, we proved that the 10 bases long PNA strand TF-PNA1K, designed to be complementary to a part of the G-rich strand of the Bcl-2 promoter sequence known as bcl2midG4, can form stable DNA-PNA complexes with its DNA target regardless the structuration of the latter in the G-quadruplex or duplex secondary structure. In the first case, we found that TF-PNA1K can bind the bcl2midG4-G4 G-quadruplex promoting the formation of DNA-PNA multimers, while in the second case we observed the formation of the bcl2midG4-ds/TF-PNA1K heterotriplex. In addition, the fact that TF-PNA1K is not released from the heterotriplex complex at temperatures lower than 40 °C at the acidic pH mimicking the one found in cancer cells microenvironment [54] supports the potential applicability of triplex-forming PNAs in the development of novel anti-Bcl-2 anticancer agents utilizable at the physiological body temperature. Conventional and accelerated MD simulations provided valuable insights into the stability and structural characteristics of the bcl2midG4-ds/TF-PNA1K heterotriplex. These findings have implications for the future design of other TF-PNAs targeting G-rich DNA sequences, as they shed light on the importance of protonation patterns, position of protonation sites, and the influence of PNAs on the DNA structure. Further studies are expected in the near future to develop effective formulations for the targeted delivery of PNAs in cancer cells [17]. Taken together, the present study strengthens our hypothesis about the development of anticancer PNA agents capable of targeting the P1 promoter of the Bcl-2 proto-oncogene regardless its structuration in the G-quadruplex or duplex arrangement.

## Data availability

All data needed to evaluate the conclusions in the paper are present in the paper and/or Supplementary Materials. Molecular models of the most representative conformations of P3 and P4 heterotriplex systems obtained by conventional and accelerated molecular dynamics have been deposited in the Zenodo repository and are freely accessible under 10.5281/zenodo.8033968.

## CRediT authorship contribution statement

**Andrea P. Falanga:** Investigation, Formal analysis, Data curation. **Antonio Lupia:** Investigation, Formal analysis, Data curation. **Lorella Tripodi:** Investigation, Formal analysis, Data curation. **Carmine M. Morgillo:** Investigation. **Federica Moraca:** Investigation, Formal analysis, Data curation. **Giovanni N. Roviello:** Writing – review & editing, Formal analysis. **Bruno Catalanotti:** Formal analysis, Data curation, Conceptualization. **Jussara Amato:** Investigation. **Lucio Pastore:** Resources, Formal analysis. **Vincenzo Cerullo:** Resources, Formal analysis, Data curation. **Stefano D'Errico:** Investigation. **Gennaro Piccialli:** Writing – review & editing, Resources. **Giorgia Oliviero:** Conceptualization. **Nicola Borbone:** Writing – original draft, Writing – review & editing, Supervision, Resources, Project administration, Funding acquisition, Formal analysis, Data curation, Conceptualization.

## Declaration of competing interest

The authors declare that they have no known competing financial interests or personal relationships that could have appeared to influence the work reported in this paper.
