# Supplementary material for: Exploring the DNA2-PNA heterotriplex formation in targeting the Bcl-2 gene promoter: A structural insight by physico-chemical and microsecond-scale MD investigation
Source: Heliyon. 2024 Jan 22;10(3):e24599. doi: 10.1016/j.heliyon.2024.e24599 (PMC10839560; doi:10.1016/j.heliyon.2024.e24599)
Supplement: Multimedia component 1 [file mmc1.docx]

**Supplementary Material**

**Exploring the DNA_2_-PNA heterotriplex formation in targeting the Bcl-2 gene promoter: a structural insight by physico-chemical and microsecond-scale MD investigation**

Andrea P. Falanga^1,#^, Antonio Lupia^1,#^, Lorella Tripodi^2^, Carmine Morgillo^1^, Federica Moraca^1^, Giovanni N. Roviello^3^, Bruno Catalanotti^1^, Jussara Amato^1^, Lucio Pastore^2^, Vincenzo Cerullo^2,4^, Stefano D’Errico^1^, Gennaro Piccialli^1^, Giorgia Oliviero^2^ and Nicola Borbone^1,^*

^1^ Dipartimento di Farmacia, Università degli Studi di Napoli Federico II, Naples, 80131, Italy

^2^ Dipartimento di Medicina Molecolare e Biotecnologie Mediche, Università degli Studi di Napoli Federico II, Naples, 80131, Italy

^3^ Istituto di Biostrutture e Bioimmagini, Consiglio Nazionale delle Ricerche, Naples, 80131, Italy

^4^ ImmunoViroTherapy Lab (IVT), Drug Research Program (DRP), Faculty of Pharmacy, University of Helsinki, 00100, Helsinki, Finland

# These authors contributed equally

* To whom correspondence should be addressed. Tel: +39 081 678521; Email: [nicola.borbone@unina.it](mailto:nicola.borbone@unina.it)


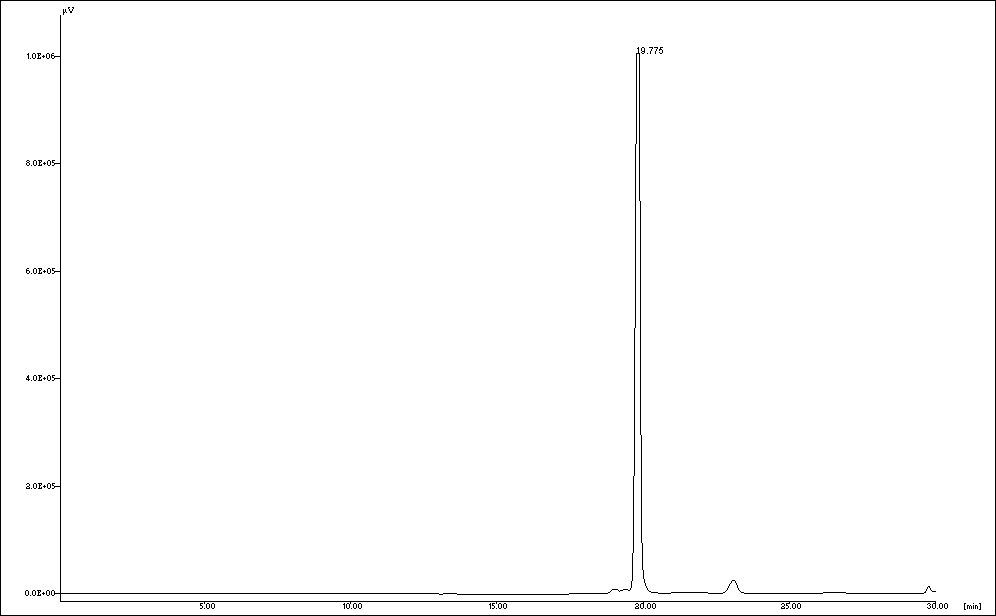


Figure S1. HPLC profile of TF-PNA6K purification by C18 reverse-phase chromatography (particle size 5 µm).

Figure S2. Unit and enhanced resolution (inset) ESI-MS (m/z) of TF-PNA1K. Calcd. for [M + 2H]^2+^ 1351.6; calcd. for [M + 3H]^3+^ 901.4.


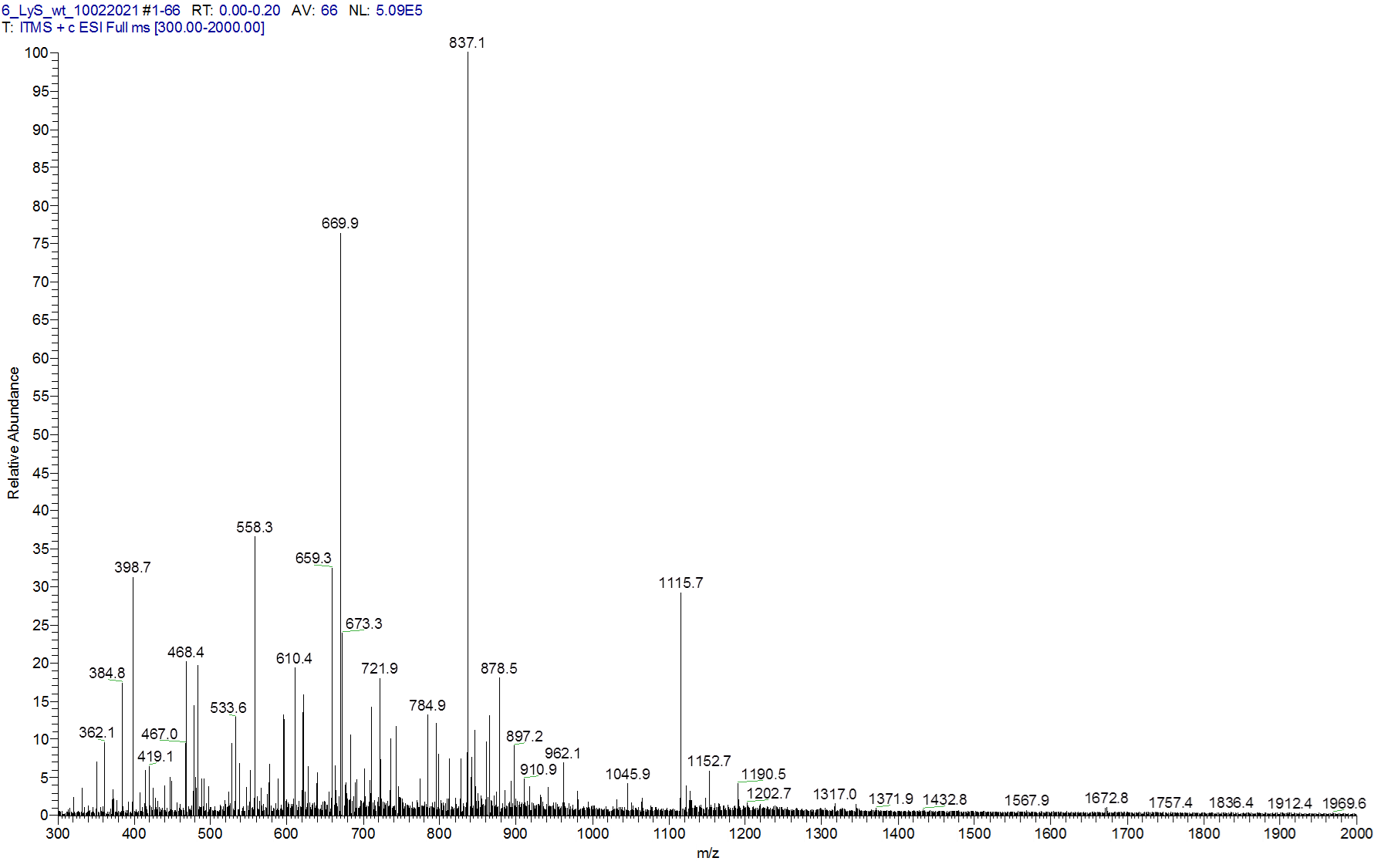


Figure S3. Unit and enhanced resolution (inset) ESI-MS (m/z) of TF-PNA6K. Calcd. for [M+3H]^3+^ 1114.9; calcd. for [M+4H]^4+^ 836.4.

Table S1. Description of the systems studied with MD simulations. PNA cytosines considered in the N3-protonated form were reported in bold red, N3-imino cytosines are in black.

| 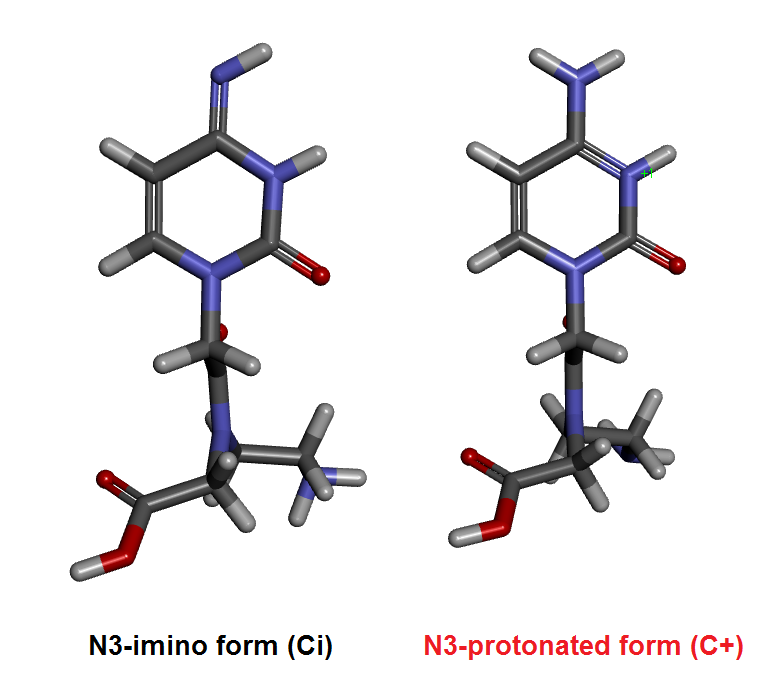 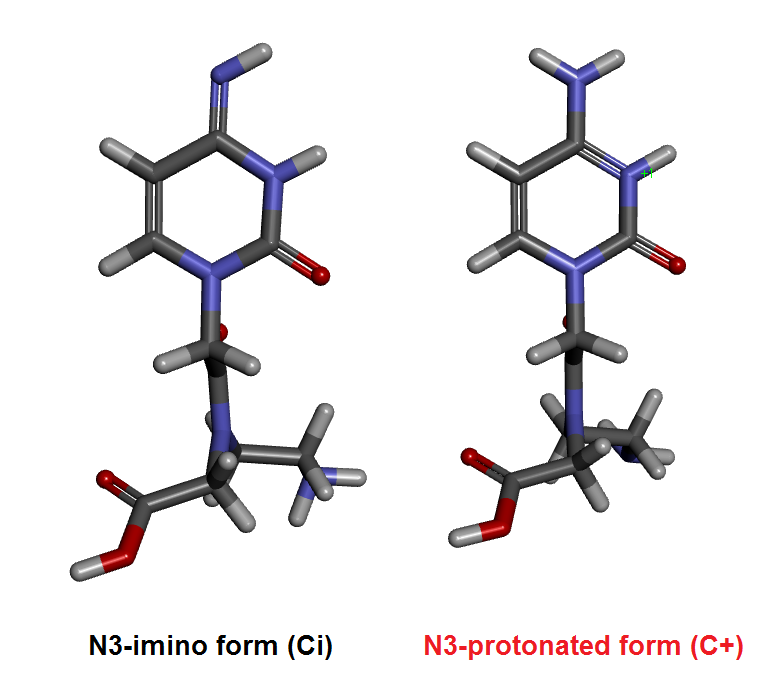 | | | |
| --- | --- | --- | --- |
| DNA1 | DNA2 | P3 | P4 |
| 5’ C_1_ | G_20_ 3’ | pC_21_-N-term | **pC_21_**-N-term |
| C_2_ | G_19_ | **pC_22_** | pC_22_ |
| C_3_ | G_18_ | pC_23_ | **pC_23_** |
| C_4_ | G_17_ | **pC_24_** | pC_24_ |
| C_5_ | G_16_ | pC_25_ | **pC_25_** |
| T_6_ | A_15_ | pT_26_ | pT_26_ |
| T_7_ | A_14_ | pT_27_ | pT_27_ |
| C_8_ | G_13_ | pC_28_ | **pC_28_** |
| C_9_ | G_12_ | **pC_29_** | pC_29_ |
| 3’T_10_ | A_11_ 5’ | pT_30_ | pT_30_ |
|  |  | Lys_31_-C-term | Lys_31_-C-term |

Table S2. Description of the minimization steps.

|  | ***Ntmin**** | ***Restraints***** | ***Cycles^§^*** | ***Ncyc^#^*** | ***Unrestrained Units*** |
| --- | --- | --- | --- | --- | --- |
| ***Step 1*** | 1 | 50 | 3000 | 1500 | Hydrogens |
| ***Step 2*** | 1 | 50 | 5000 | 2500 | Hydrogens, waters and ions |
| ***Step 3*** | 1 | 50 | 50000 | 25000 | PNA |
| ***Step 4*** | 0 | 0.5 | 20000 | - | All atoms except  OP1, P, OP2 |
| ***Step 5*** | 0 | 0.1 | 20000 | - |  |
| ***Step 6*** | 0 | 0.5 | 50000 | - |  |

*Method of Minimization: 0=*Full conjugate gradient* and 1=*steepest descent*. ******Force constant for restraint express in kcal/mol·Å^2^. ***^§^***Maximum number of minimization cycles to use per snapshot in sander. ^#^Number of steepest descent line minimization steps to perform before switching to a conjugate gradient method.

Table S3. DBSCAN Cluster populations results.

|  | S | TFr | #cl | f* | n°1^st^ | N°2^nd^ | N°3^rd^ | *f1 | *f2 | *f3 | ^§^f1 | ^§^f2 | ^§^f3 |
| --- | --- | --- | --- | --- | --- | --- | --- | --- | --- | --- | --- | --- | --- |
| cmd | P3 | 4335 | 0 | 0.72 | 997 | 1623 | 1715 | 0.5 | 0.8 | 0.8 | 1 | 1 | 1 |
|  |  | 674 | 1 | 0.11 | 674 | 0 | 0 | 0.3 | 0 | 0 | 765 | -1 | -1 |
|  |  | 105 | 2 | 0.02 | 0 | 10 | 95 | 0 | 0 | 0 | -1 | 613 | 131 |
|  | P4 | 5482 | 0 | 0.91 | 1661 | 1885 | 1936 | 0.83 | 0.94 | 0.97 | 1 | 1 | 1 |
|  |  | 261 | 1 | 0.04 | 261 | 0 | 0 | 0.13 | 0 | 0 | 459 | -1 | -1 |
| amd | P3 | 2717 | 0 | 0.90 | 902 | 977 | 838 | 0.90 | 0.98 | 0.84 | 1 | 1 | 1 |
|  |  | 99 | 1 | 0.03 | 0 | 0 | 99 | 0 | 0 | 0.1 | -1 | -1 | 335 |
|  | P4 | 2230 | 0 | 0.74 | 999 | 274 | 957 | 0.99 | 0.27 | 0.96 | 1 | 1 | 1 |
|  |  | 377 | 1 | 0.12 | 0 | 377 | 0 | 0 | 0.38 | 0 | -1 | 615 | -1 |

**S:** system. **TFr:** Total number of frames in the cluster. **#Cl**: Cluster number. ***F**: Fraction of total frames in the cluster. **NumIn1^st^**, **NumI2^nd^**, **NumI3^rd^**: Number of frames of that cluster that fall into the first, second and third trajectory, respectively. ***F**: Fraction of frames of that cluster that fall into the first, second and third trajectory, respectively. **^§^f1**: frame at which the cluster was first encountered in the first, second and third trajectory, respectively.

**
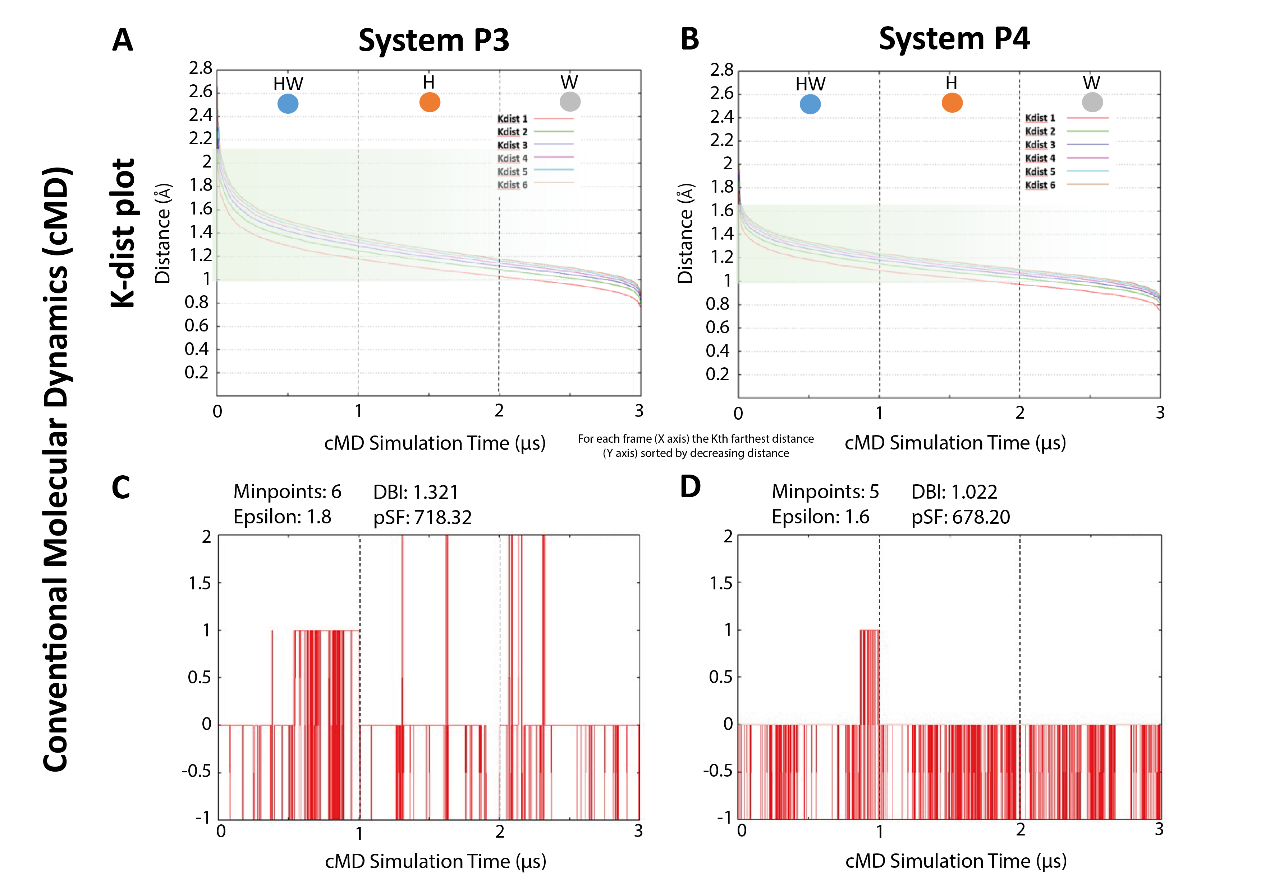
** **
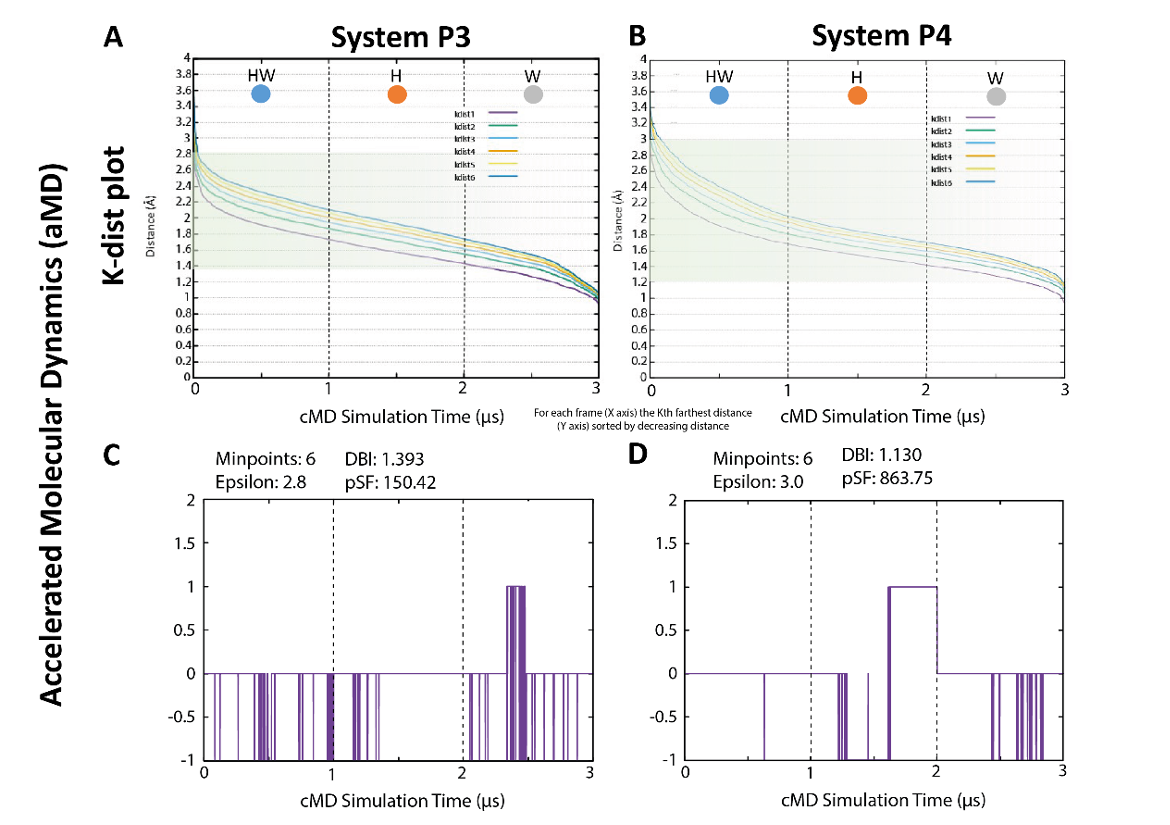
**

Figure S4. Conventional molecular dynamics (cMD) and accelerated molecular dynamics (aMD) clustering from three independent runs (HW, H, W). (A, B) K-distance plots for the DBSCAN tuning parameter and (C, D) cluster populations plot versus time, for P3 and P4 systems, respectively. The *minpoints*, *Ɛ*, *DBI* and *pSF* values are highlighted in the upper. The plots show for each frame (*X-axis*) the K-th farthest distance (*Y-axis*), sorted by decreasing distance. The analysis was performed considering all residues (1 to 32) except hydrogens.


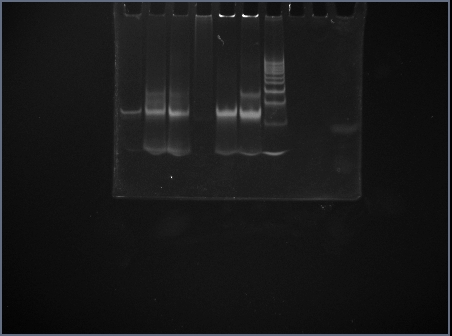


1. bcl2midG4-ds annealed at pH 5
2. bcl2midG4-ds/TF-PNA1K annealed at pH 5
3. bcl2midG4-ds/TF-PNA1K annealed at pH 5
4. bcl2midG4-ds annealed at pH 7
5. bcl2midG4-ds/TF-PNA1K annealed at pH 7
6. DNA ladder

**A**

1

2

3

4

5

6

**B**

4

3

5

2

1

6

**
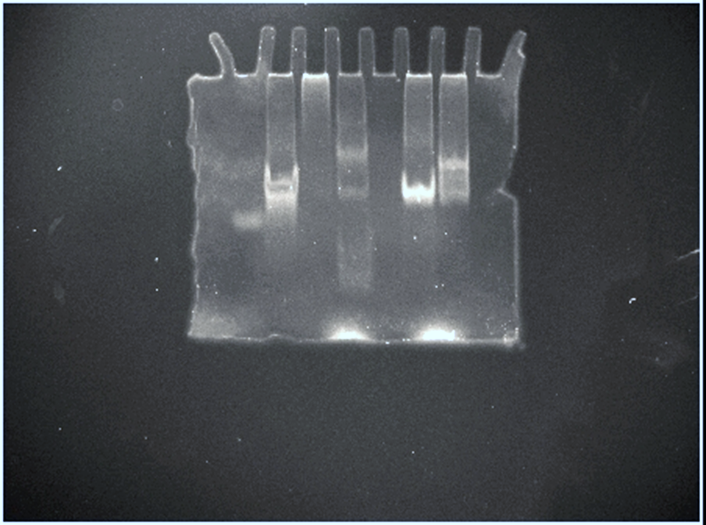
**

1. Dye
2. bcl2midG4-ds annealed at pH 5
3. bcl2midG4_G annealed at pH 5
4. bcl2midG4_G/TF-PNA1K annealed at pH 5
5. bcl2midG4-ds annealed at pH 5
6. bcl2midG4-ds/TF-PNA1K annealed at pH 5

**C**

1

2

3

4

5

6

7

8

9

10

**
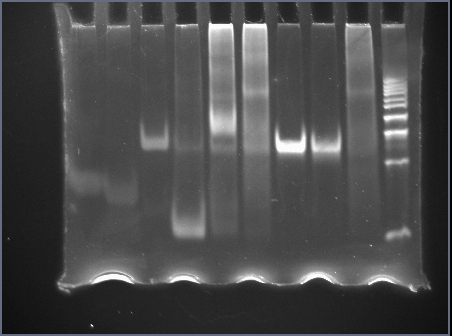
**

1. Dye
2. d(TGGGGT) annealed at pH 7 [Bioconj. Chem., 2006, 17, 889–898]
3. d(CGGTGGT) annealed at pH 7 [Nucleic Acids Res., 2011, 39, 7848–7857]
4. bcl2midG4_G annealed at pH 7
5. bcl2midG4_G/PNA1K annealed at pH 7 [Bioconj. Chem., 2019, 30, 572–582]
6. bcl2midG4_G/TF-PNA1K annealed at pH 7
7. bcl2midG4-ds annealed at pH 7
8. bcl2midG4-ds/PNA1K annealed at pH 7 [3]
9. bcl2midG4-ds/TF-PNA1K annealed at pH 7
10. DNA ladder

Figure S5. Not processed images of PAGE panels reported in Figure 4A (A; lanes 4–6), 4B (B; lanes 5 and 6), and 4C (C; lanes 4, 6, and 10). All gels were run in 1×Tris-Borate-EDTA (TBE) buffer supplemented with 30 mmol/L KCl, pH 7.0 (A and C) or pH 5.0 (B) at the constant voltage of 120 V.

Table S4. Boosting coefficients used in aMD production.

| System | | E(P) | α(P) | E(γ) | α(γ) |
| --- | --- | --- | --- | --- | --- |
| P3 | HW | -59785 | 2913 | 700 | 25.6 |
|  | H | -59786 | 2912 | 704 | 25.6 |
|  | W | -59785 | 2912 | 704 | 25.6 |
| P4 | HW | -59785 | 2913 | 700 | 25.6 |
|  | H | -59786 | 2912 | 704 | 25.6 |
|  | W | -59785 | 2913 | 704 | 25.6 |

Table S5. Analysis of helicoidal parameters. The dominant structures cluster centroid (the representative frame of the cluster analysis) of the simulated systems are compared to the canonical A-DNA and B-DNA duplex and the averaged NMR ensemble of Py-Pu-Py triplex (PDB ID:149D). Data are calculated reporting the average of the base-pairs values (excluding the terminal bases). Standard deviations are reported in brackets.

|  | **Local dimer step parameters** | | | | | | **Local helical parameters** | | | | **pP** | **Groove widths** | |
| --- | --- | --- | --- | --- | --- | --- | --- | --- | --- | --- | --- | --- | --- |
|  | ****D*x** | ***Dy** | ****Dz*** | ***τ** | ***ρ** | ***ω** | **^⸸^***d****x*** | ***η** | ****h*** | ***Ω_h_** | **^⸸^*Z_P_*** | **^⸸^MjG** | **^⸸^MnG** |
| **^a^ A-DNA** | 0.0  (0.0) | -1.4 (0.0) | 3.3 (0.0) | 0.2 (0.1) | 12.4  (0.0) | 30.3 (0.6) | -4.5 (0.1) | 22.6 (0.4) | 2.6 (0.0) | 32.7 (0.5) | 2.5 (0.0) | 18.5 (0.0) | 15.2 (0.0) |
| **^a^ B-DNA** | 0.0 (0.0) | 0.5 (0.0) | 3.4 (0.0) | 0.0 (0.0) | 1.7  (0.0) | 36.0 (0.7) | 0.5 (0.0) | 2.8 (0.1) | 3.4 (0.0) | 36.0 (0.7) | 0.5 (0.0) | 17.2 (0.0) | 11.7 (0.0) |
| **bcl2midG4-ds10** | -0.1 (0.7) | -1.1 (0.6) | 3.4 (0.2) | -0.2 (3.7) | 4.0 (5.4) | 29.6 (5.9) | -3.2 (1.8) | 8.7 (10.5) | 3.1 (0.3) | 30.5 (5.7) | 0.7 (0.2) | 21.7 (1.1) | 14.0 (0.7) |
| **P3** | -0.0  (0.4) | -1.8  (0.3) | 3.4  (0.1) | -0.7  (0.9) | 2.1  (3.1) | 27.5  (1.4) | -4.3 (0.1) | 4.2  (6.4) | 3.2  (0.2) | 27.7  (1.4) | 0.8  (0.3) | 25.9 | 12.1 |
| **P4** | -0.2  (0.4) | -1.8  (0.3) | 3.4  (0.1) | -0.5  (1.9) | 1.8  (3.6) | 27.9  (1.7) | -4.1  (0.7) | 3.6  (7.0) | -3.3  (0.2) | 28.2  (1.9) | 1.0  (0.3) | 25.0 | 13.7 |
| **149D** | -0.2 (0.1) | -0.5 (0.1) | 3.5 (0.1) | -0.8 (0.4) | 5.8 (0.7) | 30.9 (0.2) | -2.1 (0.1) | 10.9 (1.2) | 3.3 (0.1) | 31.9 (0.3) | 0.2 (0.1) | 21.3 (0.5) | 13.3 (0.4) |

^a^ Built-in 3D Dart webserver (1). ***Dx*** = Shift; ***Dy*** = Slide; ***Dz*** = Rise; **τ** = Tilt , **ρ** = Roll; **ω** = Twist; ***dx*** = *x*-displacement; ***h*** = helical rise; **Ω_h_** = helical twist and **η** = inclination; **Z_p_** = the projection of the phosphorus atom onto the *z*-axis of the dimer ‘middle frame’ (2) Values expressed in *****degrees (°) and **^⸸^**angström (Å); **pP** = Phosphorus position; major (**MjG**) and minor (**MnG**) groove.

1. van Dijk,M. and Bonvin,A.M.J.J. (2009) 3D-DART: A DNA structure modelling server. *Nucleic Acids Res.*, **37**.

2. Lu,X.-J., Shakked,Z. and Olson,W.K. (2000) A-form Conformational Motifs in Ligand-bound DNA Structures. *J. Mol. Biol.*, **300**, 819–840.


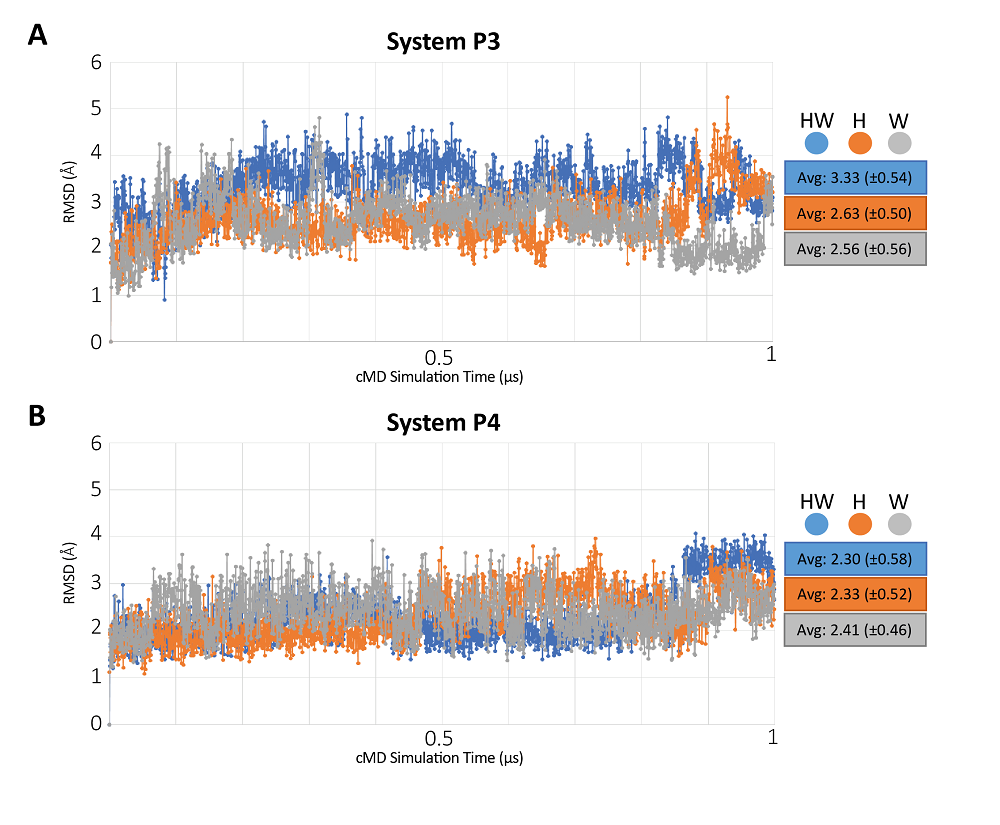


Figure S6. Three independent root mean square deviations (RMSD) of the P3 (A) and P4 (B) systems (H, W, HW), calculated on all atoms except hydrogens. The average and the standard deviations (in brackets) values are reported at the top of each graph.


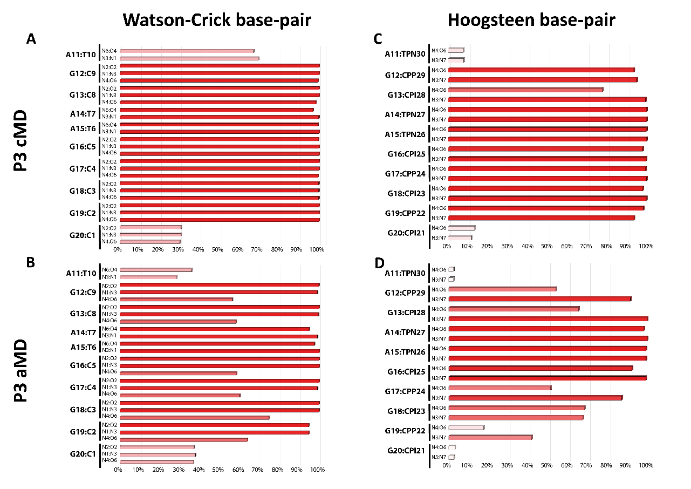


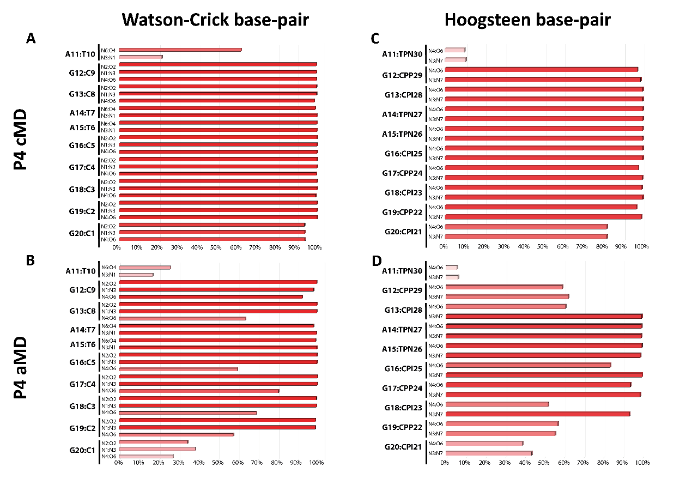


Figure S7. H-bond occupancy for the Watson–Crick and Hoogsteen's base-pairs of both P3 and P4 systems during cMD and aMD simulations. The bars plots are shaded in red according to the percentage value. Higher percentage values, reflect more interaction between the base pairs.


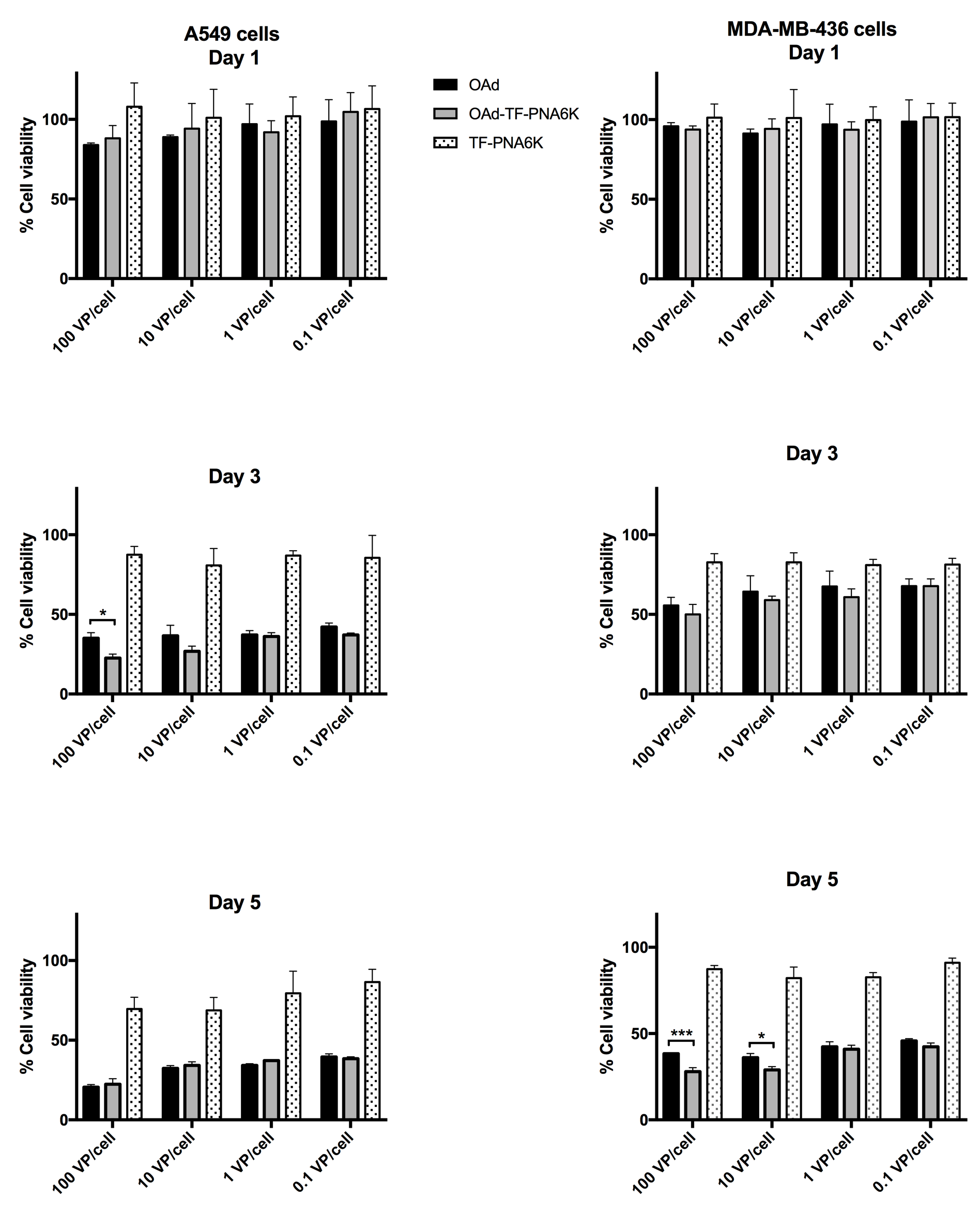


Figure S8. Viability of A549 and MDA-MB 436 cells after one, three, and five days of treatment with OAd, OAd/TF-PNA6K 1:100 (w/w), or TF-PNA6K alone. The concentrations from 100 VP/cell to 0.1 VP/cell were tested. Cell viability is reported as percentage of viable cells compared to untreated cells. Significance was assessed using the two-way ANOVA, *p<0.05, **p≤0.01, ***p<0.001, ****p<0.0001.


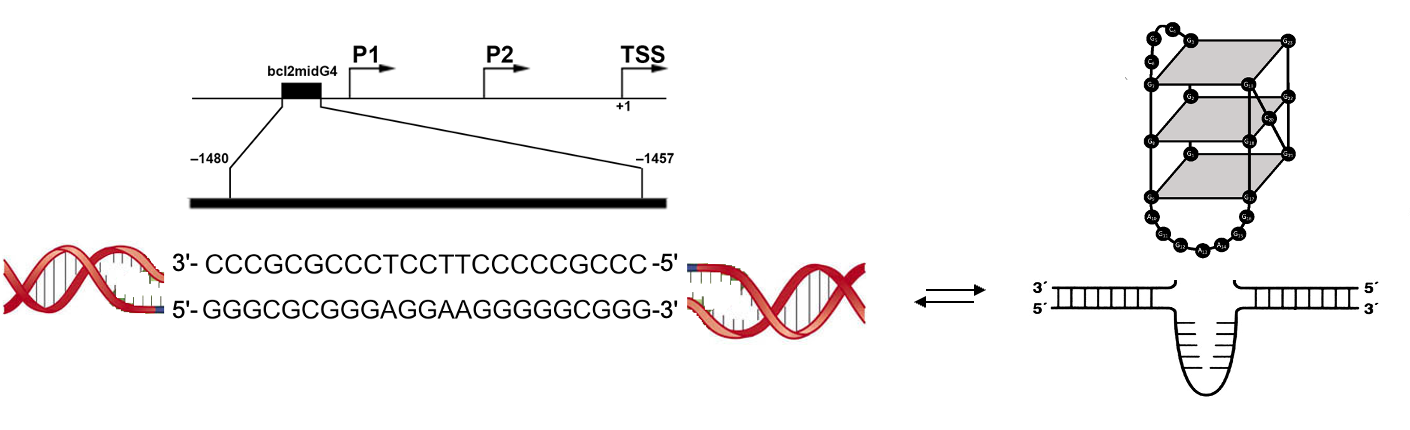


Figure S9. Schematic representation of the Bcl-2 promoter region, both in duplex and G-quadruplex folding.
